# Optimization of ultrasound-assisted extraction of polyphenols from *Mansoa alliacea* flowers and evaluation of their antioxidant, anti-inflammatory-related, and digestive enzyme inhibitory activities

**DOI:** 10.1016/j.bbrep.2026.102851

**Published:** 2026-10-31

**Authors:** Quoc Trung Nguyen, Tran Chi Linh

**Affiliations:** a Faculty of Chemistry, University of Science, Ho Chi Minh City, 700000, Viet Nam; b Vietnam National University, Ho Chi Minh City, 700000, Viet Nam; c Faculty of Medicine, College of Health Sciences, Nam Can Tho University, 168 Nguyen Van Cu Extension Street, An Binh Ward, Can Tho, 94000, Viet Nam

**Keywords:** Anti-inflammatory, Antioxidant, Box-Behnken design, Digestive enzyme inhibition, *Mansoa alliacea* flowers

## Abstract

**Background:**

*Mansoa alliacea* is traditionally used to treat inflammation, infections, and metabolic disorders. However, the phytochemical and biological potential of its flowers remains poorly characterized.

**Purpose:**

This study optimized ultrasound-assisted extraction (UAE) of polyphenols from *M. alliacea* flowers and evaluated the *in vitro* antioxidant, anti-inflammatory-related, and digestive enzyme inhibitory activities of the optimized extract.

**Methods:**

Four extraction parameters (ethanol concentration, extraction temperature, extraction time, and solid-to-solvent ratio) were initially screened by single-factor experiments. Based on these results, three key factors (temperature, time, and ethanol concentration) were selected and further optimized using a Box–Behnken design to maximize polyphenol yield. Total polyphenol content was quantified using the Folin–Ciocalteu method. UHPLC–MS profiling was conducted for tentative phytochemical characterization. The optimized extract was evaluated for *in vitro* antioxidant activity (DPPH, ABTS assays), anti-inflammatory activity (protein denaturation and RBC membrane stabilization), and digestive enzyme inhibitory activities (inhibition of *α*-amylase and *α*-glucosidase).

**Results:**

Optimal extraction conditions (60% ethanol, 79 °C, 15 min, 1:10 w/v) yielded 275.37 ± 1.55 mg GAE/g extract. UHPLC–MS analysis revealed nine major peaks, among which two compounds were provisionally assigned as luteolin-7-O-glucoside and luteolin-7-O-glucuronide. Compared with the unoptimized extract, the optimized extract exhibited markedly stronger *in vitro* biological activities, with IC_50_ values of 11.88 ± 0.28 and 15.58 ± 0.33 μg/mL in DPPH and ABTS assays, respectively; 38.77 ± 0.93 μg/mL for inhibition of protein denaturation; 30.74 ± 0.18 μg/mL for RBC membrane stabilization; and 30.94 ± 0.54 and 13.32 ± 0.19 μg/mL for α-amylase and α-glucosidase inhibition, respectively.

**Conclusion:**

Optimized UAE significantly enhanced polyphenol enrichment in the extract and improved the *in vitro* antioxidant capacity, protective effects against protein denaturation and RBC hemolysis, and carbohydrate-hydrolyzing enzyme inhibitory activities of *M. alliacea* flower extract. These findings provide preliminary scientific support for the traditional use of *M. alliacea* and suggest that its polyphenol-rich extract may serve as a promising candidate for further investigation as a functional or phytopharmaceutical ingredient.

## Highlights

• Optimized UAE increased total polyphenols 3.5-fold vs unoptimized extract.
• UHPLC–MS revealed nine peaks, two identified as luteolin derivatives.
• Luteolin-7-O-glucoside and luteolin-7-O-glucuronide were markedly enriched.
• OMAE showed stronger antioxidant, anti-inflammatory, and antidiabetic effects.
• Enrichment of luteolin conjugates improves bioactivity.

## Graphical abstract

**Figure:**
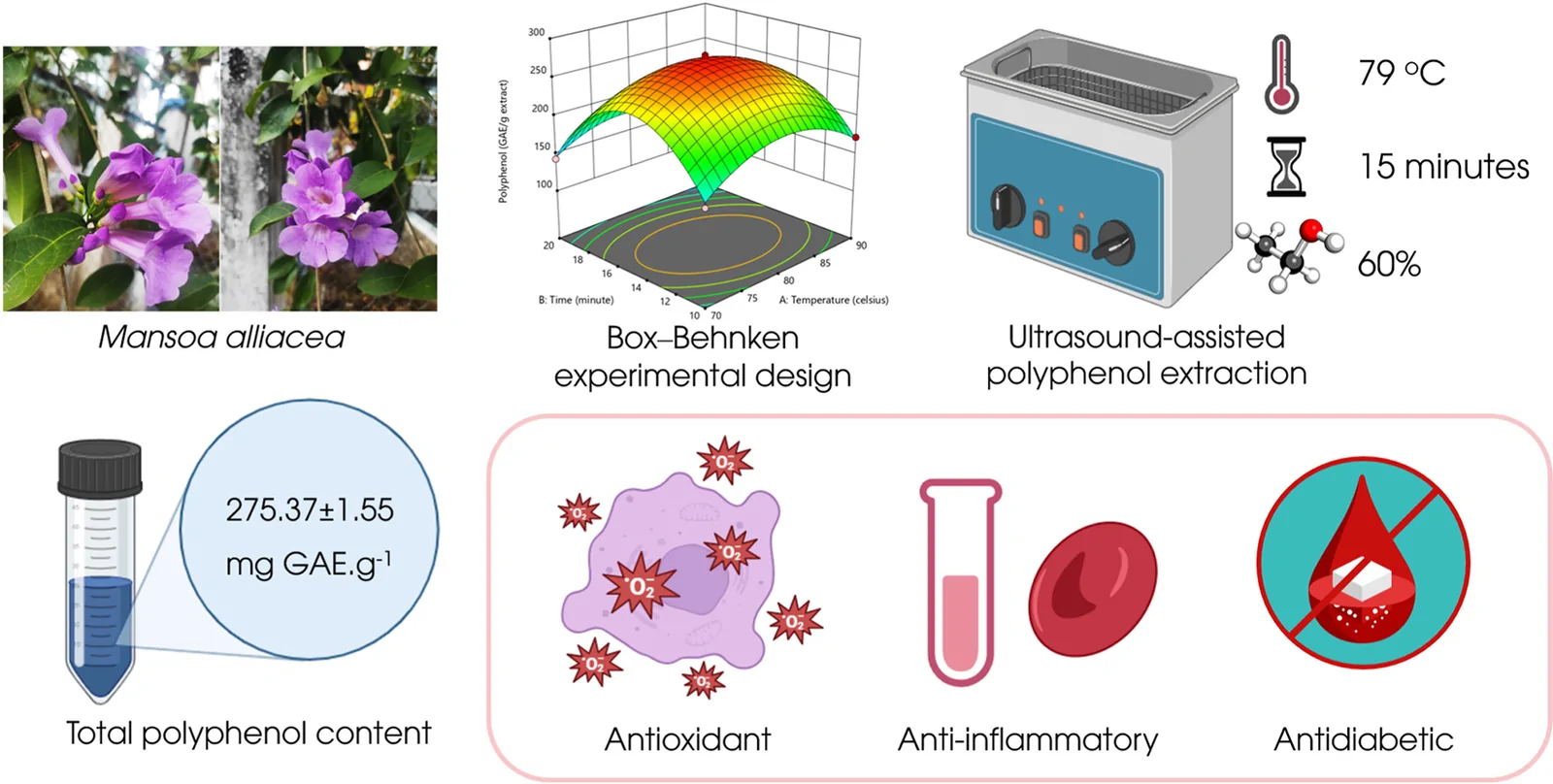

## 1 Introduction

*Mansoa alliacea* is a tropical climbing plant in the Bignoniaceae family, known for its garlic-like aroma. It is cultivated as an ornamental and used in traditional medicine for treating rheumatoid arthritis, inflammation, and pain, as well as respiratory ailments such as pneumonia, bronchitis, cough, and influenza [1]. Phytochemical investigations have identified numerous bioactive constituents in *M. alliacea*, including various organosulfur compounds (diallyl disulfide, diallyl trisulfide, alliin, allicin), sterols (*β*-sitosterol, fucosterol, stigmasterol, daucosterol), iridoids, isothiocyanates, naphthoquinones, alkaloids, saponins, flavonoids, and other polyphenols [2], [3]. The presence of these compounds, especially phenolics, is thought to underlie the plant's reported *in vitro* bioactivities, which include anticancer [4], antioxidant [5], antifungal [6], anti-inflammatory [7] and antilarval properties [8], [9].

Polyphenols are a broad class of plant secondary metabolites characterized by multiple phenolic hydroxyl groups. This class encompasses phenolic acids, stilbenes, flavonoids, tannins, and lignins. Polyphenols are among the most widely studied phytochemicals due to their high nutritional value and diverse bioactivities, including potent antioxidant, anti-inflammatory, antimicrobial, and antiviral properties [10]. Many polyphenolic compounds have been exploited in pharmaceutical formulations and functional health products [11]. Given these benefits, developing efficient methods to extract polyphenols from plant sources is of great interest for nutraceutical and medicinal applications.

Although there have been numerous studies worldwide on the phytochemistry and biological activities of *M. alliacea* leaves, relatively little attention has been paid to its flowers, especially in Vietnam. To address this gap, the present work focuses on *M. alliacea* flowers collected in Vietnam, aiming to optimize the extraction of their polyphenols and to evaluate the biological activities of the resulting extracts. Recent studies, including our previous works, have optimized the ultrasound-assisted extraction of alkaloid-rich and flavonoid-rich fractions from *M. alliacea* flowers and demonstrated promising biological activities, including antioxidant, antibacterial, and anti-obesity effects [12], [13]. However, polyphenols represent a broader and chemically distinct class of phytochemicals that includes not only flavonoids but also other phenolic constituents with potentially different extraction behaviors and biological functions. To date, no study has specifically optimized the extraction of polyphenol-rich fractions from *M. alliacea* flowers or evaluated their anti-inflammatory and antidiabetic activities in relation to polyphenol enrichment. Therefore, the present study aimed to optimize the ultrasound-assisted extraction of polyphenols from *M. alliacea* flowers and evaluate the antioxidant, anti-inflammatory, and antidiabetic properties of the optimized extract. These findings may support the development of *M. alliacea* flower extract as an ingredient in functional foods or dietary supplements targeting oxidative stress, inflammation, and glycemic control.

## 2 Materials and methods

### 2.1 Materials

Blooming *M. alliacea* flowers (Fig. 1) were collected in January 2022 from Binh Thuy District, Can Tho City, Vietnam. Botanical identification was confirmed by examining morphological characteristics, and a voucher specimen was deposited in the Biology laboratory, Medical faculty, Nam Can Tho University (storage code: CT_Man202101080010).

**Fig. 1:**
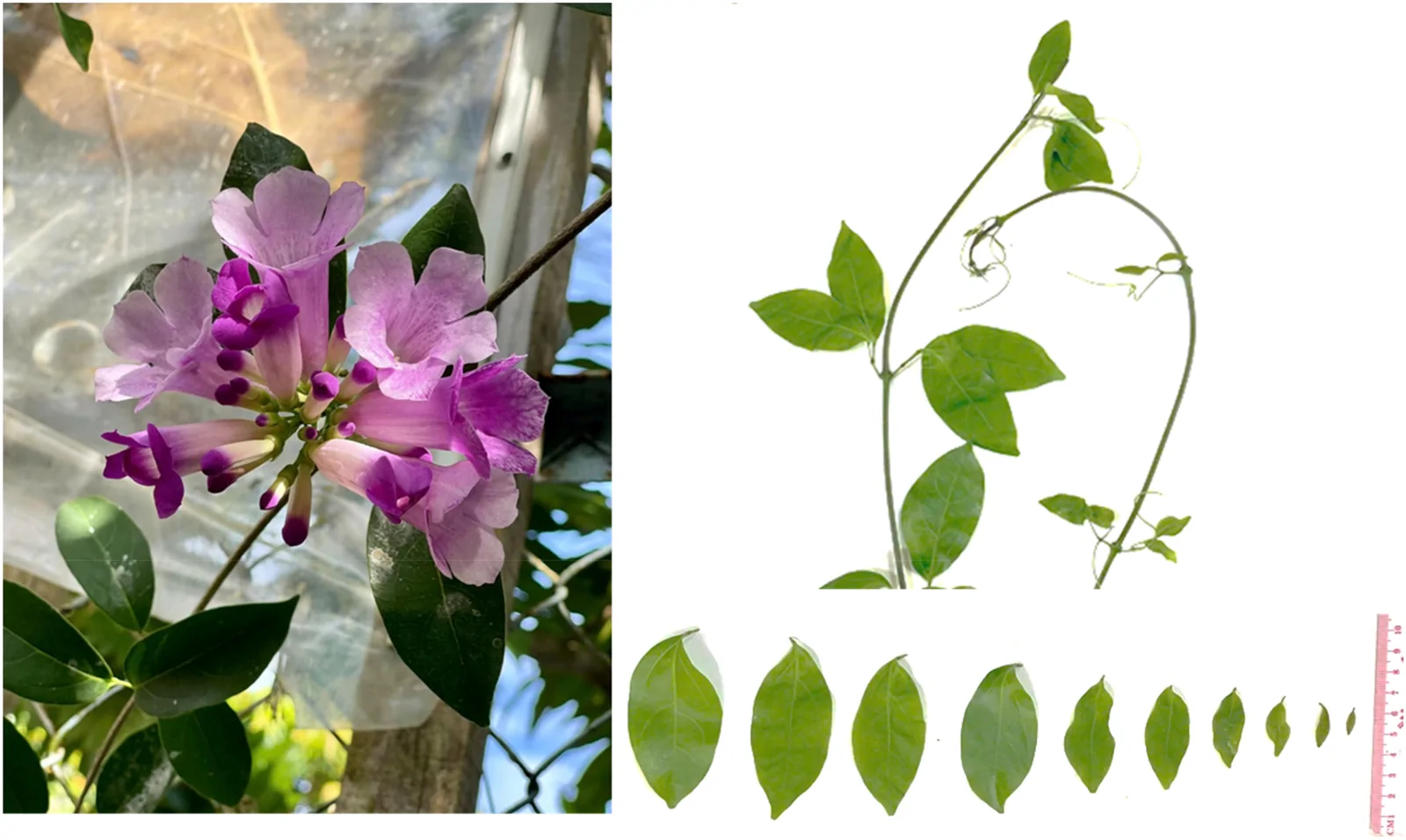
*Mansoa alliacea* (Bignoniaceae).

All chemicals and reagents used were of analytical grade. Folin–Ciocalteu phenol reagent, sodium carbonate, dimethyl sulfoxide (DMSO), potassium persulfate, and methanol were purchased from Merck (Germany). Ascorbic acid, 2,2-azino-bis(3-ethylbenzothiazoline-6-sulfonic acid) (ABTS), 2,2-diphenyl-1-picrylhydrazyl (DPPH), *α*-amylase, *α*-glucosidase, acarbose, diclofenac, and bovine serum albumin (BSA) were obtained from Sigma-Aldrich (USA).

Equipment used in the study included a drying oven (Memmert BE 200, Germany), an analytical balance (Mettler Toledo AB104-S, Switzerland), a refrigerated centrifuge (Hettich Mikro 12-24, Germany), a thermostatic incubator (Memmert, Germany), a rotary vacuum evaporator (Heidolph, Germany), and a UV-Vis spectrophotometer (Thermo Scientific Multiskan GO, Finland).

### 2.2 Preparation of flower powder and determination of moisture content

Fresh *M. alliacea* flowers (1000 g) were dried in a hot-air oven at 60 °C until constant weight. Then, dried flowers were ground into powder and sieved through a 60 mesh-fine tray to obtain 500 g of powder. The humidity of the powder was determined according to the description from Vietnam Pharmacopoeia V [14]. Afterward, it was preserved in a sealed plastic container and stored at 4 °C for experimentation. The moisture content of *M. alliacea* flower powder was approximately 7%.

### 2.3 Effects of temperature, time, ethanol concentration, and solvent ratio on polyphenol yield

Preliminary single-factor experiments were carried out to investigate the influence of four independent variables on polyphenol extraction from *M. alliacea* flower powder. In all tests, extracts were obtained by ultrasonic-assisted extraction (40 kHz), followed by filtration and solvent evaporation to dryness.

Temperature: Extractions were performed in 99.5% ethanol for 5 min at different temperatures (30, 40, 50, 60, 70, 80, and 90 °C).

Extraction time: Samples were extracted in 99.5% ethanol at 30 °C for varying durations (5, 10, 15, 20, 25, 30, and 35 min).

Ethanol concentration: Extractions were conducted at 30 °C for 5 min using ethanol–water mixtures of 40%, 50%, 60%, 70%, 80%, 90%, and 99.5% (v/v).

Solid-to-solvent ratio: Extractions were carried out at 30 °C for 5 min using 99.5% ethanol at different ratios (w/v) of 1:10, 1:15, 1:20, 1:25, 1:30, 1:35, and 1:40.

The yield of polyphenols (mg of gallic acid equivalents per g extract) was measured for all samples (Section 2.5). These single-factor tests identified the principal factors and appropriate ranges to be used in the subsequent optimization experiments (Section 2.4).

### 2.4 Extracting polyphenol-rich extract from *M. alliacea* flowers

Based on the preliminary results (Section 2.3), three key factors were selected for optimization: extraction temperature (A), extraction time (B), and ethanol concentration (C). The solid-to-solvent ratio was maintained at 1:10 (w/v), since variations in ratio showed no significant effect on yield. A Box–Behnken response surface design (Design Expert v11.0 software) was applied to determine the optimal combination of A, B, and C for maximizing polyphenol content. Each independent variable was examined at three levels (coded as −1, 0, +1). The experimental design consisted of 17 runs, comprising 12 distinct experimental conditions and five replicates at the center point (80 °C, 15 min, and 60% ethanol, v/v). Table 1 lists the coded factor levels and the experimental and predicted total polyphenol contents for all trials. After conducting the experiments per the design, the total polyphenol content (response variable, see Section 2.5 for assay) was measured for each run. A second-order polynomial model was fitted to the data to describe the relationship between total polyphenol yield (Y) and the three factors. In this study, total polyphenol content expressed as mg GAE/g extract was selected as the optimization response variable to prioritize the enrichment of polyphenols in the obtained extract rather than the overall extraction mass yield. The optimal extraction conditions predicted by the model were verified by confirmation experiments.

**Table 1:** Independent variables, coded levels, and total polyphenol content (experimental and predicted) for the Box–Behnken design.

No.	Independent variables			Total polyphenol content (mg GAE/g extract)	
	A (^○^C)	B (minutes)	C (%, v/v)	Experimental	Predicted
1	70	10	60	157.04 ± 1.81	163.67
2	90	10	60	172.30 ± 1.45	170.12
3	70	20	60	143.11 ± 3.22	145.29
4	90	20	60	129.00 ± 2.22	122.37
5	70	15	50	191.98 ± 2.35	188.70
6	90	15	50	174.74 ± 1.77	180.27
7	70	15	70	224.48 ± 1.57	218.95
8	90	15	70	207.62 ± 1.63	210.91
9	80	10	50	167.68 ± 1.25	164.33
10	80	20	50	121.32 ± 1.80	122.43
11	80	10	70	187.05 ± 1.99	185.94
12	80	20	70	158.36 ± 1.74	161.71
13	80	15	60	275.71 ± 2.05	274.80
14	80	15	60	273.04 ± 0.20	274.80
15	80	15	60	279.02 ± 0.18	274.80
16	80	15	60	270.18 ± 0.22	274.80
17	80	15	60	276.06 ± 2.57	274.80

Preparation of optimized and control extracts: The extraction condition that maximized polyphenol yield was identified from the response surface analysis. An extract was prepared under these optimal conditions and is hereafter referred to as the optimized *M. alliacea* extract (OMAE). For comparison, a control extract was prepared under non-optimized (“baseline”) conditions: 99.5% ethanol, 30 °C, 5 min ultrasonication, 1:10 w/v ratio. This unoptimized *M. alliacea* extract is referred to as UMAE. Both OMAE and UMAE were dried and used for subsequent biological assays described below.

### 2.5 Determination of the total polyphenol content

The total polyphenol content in each sample was determined with the Folin-Ciocalteu method, according to previous methods [15]. Briefly, 0.5 mL of *M*. *alliacea* flower extract (200 μg/mL in methanol), 0.5 mL deionized water, 0.5 mL Folin-Ciocalteu (20%) reagent were mixed in a test tube. After 5 min, 0.5 mL of sodium carbonate (10%) was added, vortexed, and incubated at 40 °C for 20 min. Absorbance was then measured at 765 nm (at room temperature) using the Multiskan GO spectrophotometer. Gallic acid standards were analyzed similarly, and total polyphenols in samples were expressed as milligrams of gallic acid equivalents per gram of dried extract (mg GAE/g extract).

### 2.6 UHPLC–MS conditions

Chromatographic analysis was performed on a UHPLC Ultimate 3000 system (Thermo Scientific/Dionex, USA) coupled to an MSQ Plus single-quadrupole mass spectrometer equipped with a diode-array detector (DAD). Separation was achieved on a Phenomenex Luna C18 (2) column (150 × 4.6 mm i. d., 3 μm) protected by a guard cartridge of the same material. The column temperature was maintained at 30 °C. The mobile phases were: A, H_2_O; B, MeOH; C, 0.1% (v/v) formic acid in H_2_O; and D, acetonitrile. The gradient program was: 0.0 min, 98% C/2% D; linear to 100% D at 20.0 min; then held at 100% D until 27.0 min (stop run). The flow rate was 0.5 mL/min, and the injection volume was 5 μL. Samples were dissolved in MeOH–H_2_O (50:50, v/v), filtered through a 0.22 μm PTFE membrane, and equilibrated in the autosampler prior to injection. The DAD monitored at 280 nm (bandwidth 4 nm) and simultaneously recorded full UV spectra from 200 to 400 nm for compound characterization.

Mass spectrometric detection was carried out using an electrospray ionization (ESI) source in negative ion mode. The probe temperature was set to 400 °C, the spray voltage to 3.5 kV, sheath gas (nitrogen) pressure to ∼70 psi, and auxiliary gas flow to ∼15 psi. The instrument was operated in full-scan mode over *m*/*z* 190–1500 with a scan time of 0.5 s per spectrum. Mass calibration was checked daily with the manufacturer's standard calibration solution.

Data acquisition and processing were performed with Chromeleon software version 7.2.4 (Thermo Scientific). Compound identification was tentatively based on comparison of retention times, UV spectra, and experimental mass spectra with literature data and, where available, authentic reference standards. Peaks were interpreted according to the presence of deprotonated molecular ions ([M − H]^-^) and characteristic in-source fragment ions, together with UV–Vis absorption maxima. For quality assurance, blank and replicate injections were included to confirm the absence of carry-over and to assess system repeatability.

### 2.7 In vitro antioxidant activity assays

The antioxidant capacity of the extracts was evaluated using two stable free radical scavenging assays, DPPH and ABTS^•+^, following established protocols [16], [17]. In each assay, the activity of OMAE and UMAE was compared to a positive control (ascorbic acid). DMSO (10%) in the reaction medium served as the negative control.

For the DPPH assay, 50 μL of sample (OMAE, UMAE, or ascorbic acid at various concentrations) was added to 1.95 mL of DPPH solution (0.1 mM in methanol, giving an absorbance about 0.7 ± 0.01 at 517 nm). The mixture was incubated in the dark at 37 °C for 30 min. The decrease in absorbance at 517 nm was recorded against a blank.

For the ABTS assay, ABTS radical cation (ABTS^•+^) was produced by mixing 7 mM ABTS with 2.45 mM potassium persulfate (1:1, v/v) and allowing the solution to stand in the dark for 12–16 h. The ABTS^•+^ stock was then diluted with ethanol to an absorbance of 0.70 ± 0.01 at 734 nm. In the assay, 300 μL of sample was mixed with 3.0 mL of the ABTS^•+^ working solution and incubated at 37 °C for 6 min. Absorbance at 734 nm was measured.

For both assays, the percentage of free radical scavenging was calculated using the formula:

$$\text{Scavenging}\;\left(\%\right)=\frac{A_{control}-A_{\text{sample}}}{A_{\text{control}}}\times 100$$

Where: A_control_ is the absorbance of the negative control (DMSO only) and A_sample_ is the absorbance in the presence of the extract or standard. Lower absorbance indicates higher radical scavenging ability. Each experiment was performed using three independent experimental replicates, and each sample within an experiment was measured in triplicate. The concentration of sample required to scavenge 50% of the radicals (IC_50_) was determined from the dose–response curves.

### 2.8 *In vitro* anti-inflammatory activity assays

The anti-inflammatory potential of the extracts was assessed by two *in vitro* methods: inhibition of protein (albumin) denaturation and stabilization of red blood cell (RBC) membranes, as described by Yesmin et al. [18] with minor modifications. In these assays, OMAE and UMAE were evaluated alongside a non-steroidal anti-inflammatory drug (diclofenac) as a positive control. A 10% DMSO solution served as the negative control.

Protein denaturation inhibition: A reaction mixture containing 3.8 mL of phosphate buffer (pH 7.4), 1.0 mL of sample (OMAE or UMAE at various concentrations or diclofenac), and 0.2 mL of 1% w/v BSA solution was prepared. The mixture was incubated at 37 °C for 15 min. Protein denaturation was then induced by heating the mixture at 70 °C for 15 min. After cooling, the turbidity was measured at 660 nm. The percentage inhibition of protein denaturation was calculated relative to the negative control (buffer + BSA without any inhibitor), where reduced turbidity (aggregation) indicates protection against denaturation.

Red blood cell membrane stabilization: Fresh whole blood was collected from healthy albino rats under protocols approved by the Institutional Ethics Committee in Biomedical Research of Nam Can Tho University (Approval No. 40YSH/PCT-HĐĐĐ), as part of the research project entitled “Screening of medicinal plants with potential to support the prevention and treatment of disorders related to metabolic syndrome and oxidative stress using animal models”. The collected blood was centrifuged at 3000 rpm for 10 min. The supernatant was discarded, and the RBC pellet was washed three times with normal saline. The washed erythrocytes were resuspended in saline to 10% (v/v). In the assay, 1.0 mL of 10% RBC suspension was mixed with 1.0 mL of sample (OMAE or UMAE at various concentrations or diclofenac). The mixtures were incubated at 56 °C for 30 min, then cooled to room temperature and centrifuged at 2500 rpm for 5 min. The absorbance of the supernatant was measured at 540 nm to assess the degree of hemolysis. The percentage stabilization of RBC (protection against hemolysis) was calculated by comparing the absorbance of the sample-treated blood to that of the negative control (saline + DMSO with RBCs, representing 100% hemolysis).

*The in vitro* anti-inflammatory efficacy of both methods was calculated based on the following formula:

$$\text{Inhibition}\;\left(\%\right)=\frac{B_{\text{control}}-B_{\text{sample}}}{B_{\text{control}}}\times 100$$

Where: B_control_ is the absorbance of the negative control and B_sample_ is the absorbance in the presence of the extract or diclofenac. All assays were conducted using three independent experimental replicates, with each sample measured in triplicate, and IC_50_ values (extract concentration required to achieve 50% inhibition of denaturation or hemolysis) were obtained from concentration–response curves.

### 2.9 *In vitro* digestive enzyme inhibitory activity assays

The inhibitory activities of the extracts against α-amylase and α-glucosidase were evaluated according to the method of Sagbo et al. [19], with minor modifications. In all assays, OMAE and UMAE were tested at various concentrations and compared with acarbose (positive control). A 10% DMSO solution served as the negative control.

α-Amylase inhibition assay: Sample solutions of OMAE or UMAE (various concentrations in phosphate buffer, pH 7.0) were pre-incubated with 5 μg of α-amylase (from porcine pancreas, 3 U/mL) in a total volume of 250 μL at 37 °C for 10 min. The reaction was initiated by adding 250 μL of 1% soluble starch solution (prepared in phosphate buffer). After incubation at 37 °C for 30 min, the reaction was terminated by adding 100 μL of 1 M HCl. Subsequently, 100 μL of iodine–potassium iodide solution was added, and absorbance was measured at 580 nm. Higher absorbance indicates greater enzyme inhibition (due to more residual starch). The *α*-amylase suppression efficiency was calculated with the following formula:

$$\text{Inhibition}\;\left(\%\right)=100-\left[\left(\frac{C_{blank}-C_{sample}}{C_{blank}-C_{control}}\right)\times 100\right]$$

Where: C_blank_ is the absorbance of the reaction mixture without enzyme (representing intact substrate, 100% unhydrolyzed starch). C_sample_ is the absorbance of the reaction mixture containing both enzyme and the sample extract. C_control_ is the absorbance of the reaction mixture without sample extract (representing maximum enzymatic starch hydrolysis).

α-Glucosidase inhibition assay: In a 1.5 mL reaction mixture, 0.5 mL of extract sample (or acarbose) was mixed with 0.24 mL of α-glucosidase solution (1 U/mL from *Saccharomyces cerevisiae*) in phosphate buffer (pH 7.0) and pre-incubated at 37 °C for 20 min. The reaction was started by adding 40 μL of 5 mM *p*-nitrophenyl-α-d-glucopyranoside (pNPG) substrate, followed by a further 10-min incubation at 37 °C. The reaction was stopped by adding 0.75 mL of 0.2 M Na_2_CO_3_, and the amount of released p-nitrophenol was determined spectrophotometrically at 405 nm. The *α*-glucosidase suppression efficiency was calculated with the formula:

$$\text{Inhibition}\;\left(\%\right)=\frac{D_{\text{control}}-D_{\text{sample}}}{D_{\text{control}}}\times 100$$

Where: D_control_ is the absorbance of the negative control (reaction mixture with buffer instead of sample, representing maximum enzyme activity). D_sample_ is the absorbance of the reaction mixture containing the test sample (or acarbose). For both enzyme assays, experiments were performed using three independent experimental replicates, and each concentration was analyzed in triplicate. The IC_50_ values for *α*-amylase and *α*-glucosidase inhibition were determined by plotting the inhibition percentages against the inhibitor concentrations.

### 2.10 Statistical analysis

All experiments were conducted using three independent biological replicates (n = 3), each prepared from a separately extracted batch of flower powder. For each biological replicate, measurements were performed in triplicate as technical replicates to ensure analytical precision. Technical replicates were averaged before statistical analysis and were not considered independent statistical replicates.

The half-maximal inhibitory concentration (IC_50_) values were estimated by linear regression analysis of the concentration-response data using the equation y = ax + b, where x represents the concentration of the extract or standard on a linear scale and y represents the corresponding percentage of inhibition within the linear dynamic range. All concentration-response curves showed a coefficient of determination (R^2^) greater than 0.98, as presented in Table 4.

Data are expressed as mean ± standard deviation (SD) based on the three independent biological replicates. Statistical differences among groups were assessed using one-way analysis of variance (ANOVA), followed by Tukey's honestly significant difference (HSD) post hoc test, using Minitab 16.0 software (Minitab Inc., State College, PA, USA). A p-value of less than 0.05 was considered statistically significant.

For the *in vitro* screening assays, a statistically non-significant difference (p > 0.05) was interpreted cautiously as indicating comparable *in vitro* inhibitory activity under the tested experimental conditions and was not taken to imply therapeutic equivalence.

For the Box–Behnken optimization, Design-Expert 11.0 software (Stat-Ease Inc., Minneapolis, MN, USA) was used to generate the experimental design, fit the second-order polynomial model, and construct response surface plots. Model adequacy and predictive performance were evaluated based on the coefficient of determination (R^2^), adjusted R^2^, coefficient of variation (CV), and lack-of-fit test. The significance of linear, quadratic, and interaction terms was assessed using ANOVA.

## 3 Results and discussion

### 3.1 Influence of singular factors on polyphenol extraction

*Effect of temperature:* Extraction temperature had a pronounced effect on polyphenol yield. As the temperature increased from 30 °C to 80 °C, the total polyphenol content of the *M. alliacea* flower extract rose from 117.77 ± 2.10 to 151.78 ± 2.27 mg GAE/g extract (Fig. 2A). However, a further increase to 90 °C caused a significant decrease in yield (145.12 ± 0.99 mg GAE/g extract, *p* < 0.05). Thus, the highest yield was obtained at 80 °C, with a decline at 90 °C, likely due to thermal degradation of polyphenolic compounds at high temperatures. Excessive heat can decompose heat-labile phenolics and reduce the efficiency of extraction [20].

**Fig. 2:**
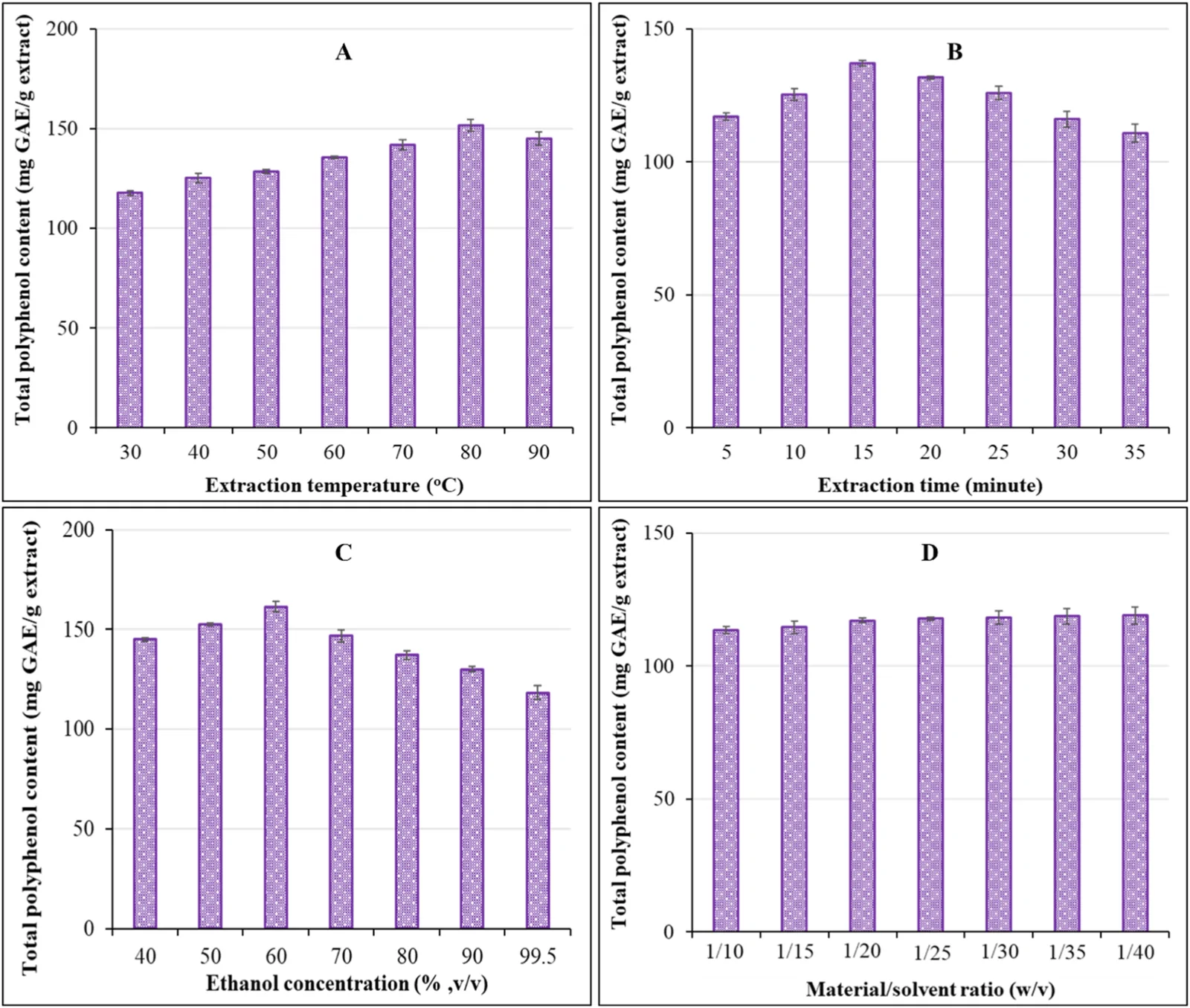
Polyphenol content of *Mansoa alliacea* extracts under the influence of single factors: temperature (A), extraction time (B), ethanol concentration (C), and material/solvent ratio (D). Data are presented as mean ± SD of three independent biological replicates (n = 3), with each measurement performed in technical triplicates. Error bars denote the standard deviation of biological replicates.

*Effect of extraction time:* Ultrasonication time also influenced polyphenol content in the resulting extract (Fig. 2B). Yield increased from 117.06 ± 0.60 mg GAE/g extract at 5 min to 137.05 ± 1.15 mg GAE/g extract at 15 min. Prolonging the sonication beyond 15 min did not further increase yield; in fact, the polyphenol content began to decline slightly after 20 min. This suggests that an extraction time around 10–15 min is sufficient, while longer exposure may lead to oxidative or ultrasonic degradation of phenolics [21], [22]. Previous studies have noted that excessively long ultrasonication can break down polyphenol compounds, diminishing the yield of intact phenolics [22].

*Effect of ethanol concentration:* The solvent's ethanol–water ratio markedly affected extraction efficiency (Fig. 2C). A moderate ethanol content was optimal: increasing ethanol concentration from 40% to 60% (v/v) raised the polyphenol yield from 145.01 ± 0.98 to 161.41 ± 2.53 mg GAE/g extract. Concentrations above 60% ethanol, however, led to a decrease in yield (146.68 ± 2.96 mg GAE/g extract at 99.5% ethanol, nearly equivalent to pure ethanol). This trend reflects the polarity requirements for extracting polyphenols. A mixture of water and ethanol provides a medium polarity solvent, which is more effective for polyphenolic compounds than either water alone (highly polar) or absolute ethanol (non-polar). Binary solvents like 60% ethanol likely optimize the solubility of a broad range of polyphenols [23]. Beyond this optimum, further increasing ethanol reduces the polarity too much, decreasing extraction of more polar phenolics.

*Effect of solid-to-solvent ratio:* Within the tested range (1:10 to 1:40 w/v), the material-to-solvent ratio had a comparatively minor effect on the polyphenol yield (Fig. 2D). The yield showed a slight upward trend as the solvent volume increased (from 1:10 to 1:40), but these changes were not statistically significant (*p* > 0.05). Similar observations have been reported by Dao et al. [24], who noted that beyond a certain threshold, using more solvent does not significantly enhance extraction. Excess solvent may dissipate ultrasonic energy and create a less efficient extraction environment, resulting in diminishing returns [24]. Therefore, a 1:10 ratio was deemed sufficient and economically favorable, as it uses the least solvent without sacrificing yield.

From the single-factor experiments above (Fig. 2), authors identified temperature, extraction time, and ethanol concentration as the principal factors affecting polyphenol enrichment in the extracts from *M. alliacea* flowers. The preliminary optimal ranges observed were: temperature around 70–90 °C, time around 10–20 min, and ethanol concentration around 50–70% v/v. The solid–solvent ratio showed no significant effect beyond 1:10, so the lowest ratio (1:10) was chosen for further experiments. These findings provided the basis for the response surface optimization using Box–Behnken design.

### 3.2 Optimization of the extraction process (Box–Behnken results)

To statistically optimize the extraction conditions for total polyphenols, a Box–Behnken response surface design was applied focusing on three independent variables: extraction temperature (A), extraction time (B), and ethanol concentration (C). The solid-to-solvent ratio was held constant at 1:10 (w/v) based on preliminary experiments and therefore was not included as a factor in the design. The experimental matrix consisted of 15 unique combinations plus replicates of the center point, totaling 17 runs. Table 1 lists the coded factor levels and the experimental and predicted total polyphenol contents for all trials. After conducting the experiments according to the design, the total polyphenol content (response variable; see Section 2.5) was measured for each run. A second-order polynomial model was fitted to the data to describe the relationship between total polyphenol yield (Y) and the three factors. The optimal extraction conditions predicted by the model were verified by confirmation experiments. The results indicated that certain combinations of conditions yielded notably higher polyphenol levels. In particular, runs 13 through 17 (which included center and high-level combinations) gave higher yields than other runs. Among the 17 experimental runs included in the Box-Behnken design, Run 15, corresponding to one of the center-point replicates at 80 °C, 15 min, and 60% ethanol, produced the highest experimentally observed polyphenol content within the design matrix (279.02 ± 0.18 mg GAE/g extract). This experimental result represents the highest value obtained at the specific design points tested. In contrast, the response surface model identified a different combination of extraction conditions as the predicted optimum through mathematical optimization.

The following second-order polynomial equation was obtained to describe the total polyphenol content (Y) as a function of temperature (A), time (B), and ethanol concentration (C):

$$Y=–4441.28+68.40\times A+102.47\times B+40.24\times C\;–\;0.15\times A\times B+0.0009\times A\times C+0.09\times B\times C\;–\;0.42\times A^{2}\;–\;3.31\times B^{2}\;–\;0.33\times C^{2}\text{.}$$

This regression model suggests positive linear effects of all three factors (A, B, C) on polyphenol yield, and it includes small interaction terms (A×B, A×C, B×C) and larger negative quadratic terms, indicating curvature in the response surface. In the fitted quadratic model, the coefficients of the quadratic terms are all negative, consistent with the existence of an optimum point within the factor ranges.

The ANOVA results demonstrated that the quadratic model was statistically significant (p < 0.05), supporting the suitability of the response surface methodology for optimization of the extraction process. The ANOVA results (Table 2) showed a lack-of-fit p-value of 0.0563, indicating no statistically significant lack of fit relative to the pure error (p > 0.05). However, because this value was close to the conventional significance threshold of 0.05, the result should be interpreted cautiously. Overall, the quadratic polynomial model adequately described the experimental responses within the investigated factor ranges, with no statistically significant evidence of model inadequacy. The coefficient of determination (R^2^ = 0.9949) and adjusted R^2^ (0.9884) indicated a high degree of agreement between the model predictions and the experimental observations within the tested factor space. Nevertheless, a high R^2^ primarily reflects the model's goodness of fit to the experimental data and does not by itself establish external robustness or generalizability beyond the investigated ranges. The low coefficient of variation (CV = 2.98%) further indicated acceptable experimental precision within the design domain.

**Table 2:** Analysis of correlation coefficients (ANOVA) of factors on polyphenol content.

Sources	Sum of squares	D_f_	Mean square	F-value	*p*-value
**Model**	49153.95	9	5461.55	153.08	< 0.0001
A-Temperature	135.71	1	135.71	3.80	0.0921
B-Time	2187.25	1	2187.25	61.31	0.0001
C-Ethanol concentration	1854.10	1	1854.10	51.97	0.0002
AB	215.65	1	215.65	6.04	0.0436
AC	0.0361	1	0.0361	0.0010	0.9755
BC	78.06	1	78.06	2.19	0.1826
A^2^	7310.58	1	7310.58	204.91	< 0.0001
B^2^	28846.48	1	28846.48	808.55	< 0.0001
C^2^	4705.11	1	4705.11	131.88	< 0.0001
**Residual**	249.74	7	35.68		
Lack of Fit	205.07	3	68.36	6.12	0.0563
Pure Error	44.67	4	11.17	N = 17	CV = 2.98%
**Cor Total**	49403.68	16	R^2^ = 0.9949	R^2^_Adj_ = 0.9884	R^2^_Pre_ = 0.9321

The linear effects of extraction time and ethanol concentration were statistically significant (p < 0.05), whereas the linear effect of extraction temperature was not significant (p = 0.0921). However, the quadratic terms of all three factors showed significant effects on polyphenol content (p < 0.05), as presented in Table 2. Among the interaction terms, the temperature × time interaction (A×B) had a significant influence on yield (*p* < 0.05), suggesting that the optimal extraction time depends somewhat on the temperature used. In contrast, the interactions between temperature and ethanol (A×C) and between time and ethanol (B×C) were not significant (*p* > 0.05). These interaction trends can be visualized in the three-dimensional response surface plots (Fig. 3), illustrating how varying two factors together affects the polyphenol yield while the third factor is held at its center level. The response surfaces show a clear optimum region.

**Fig. 3:**
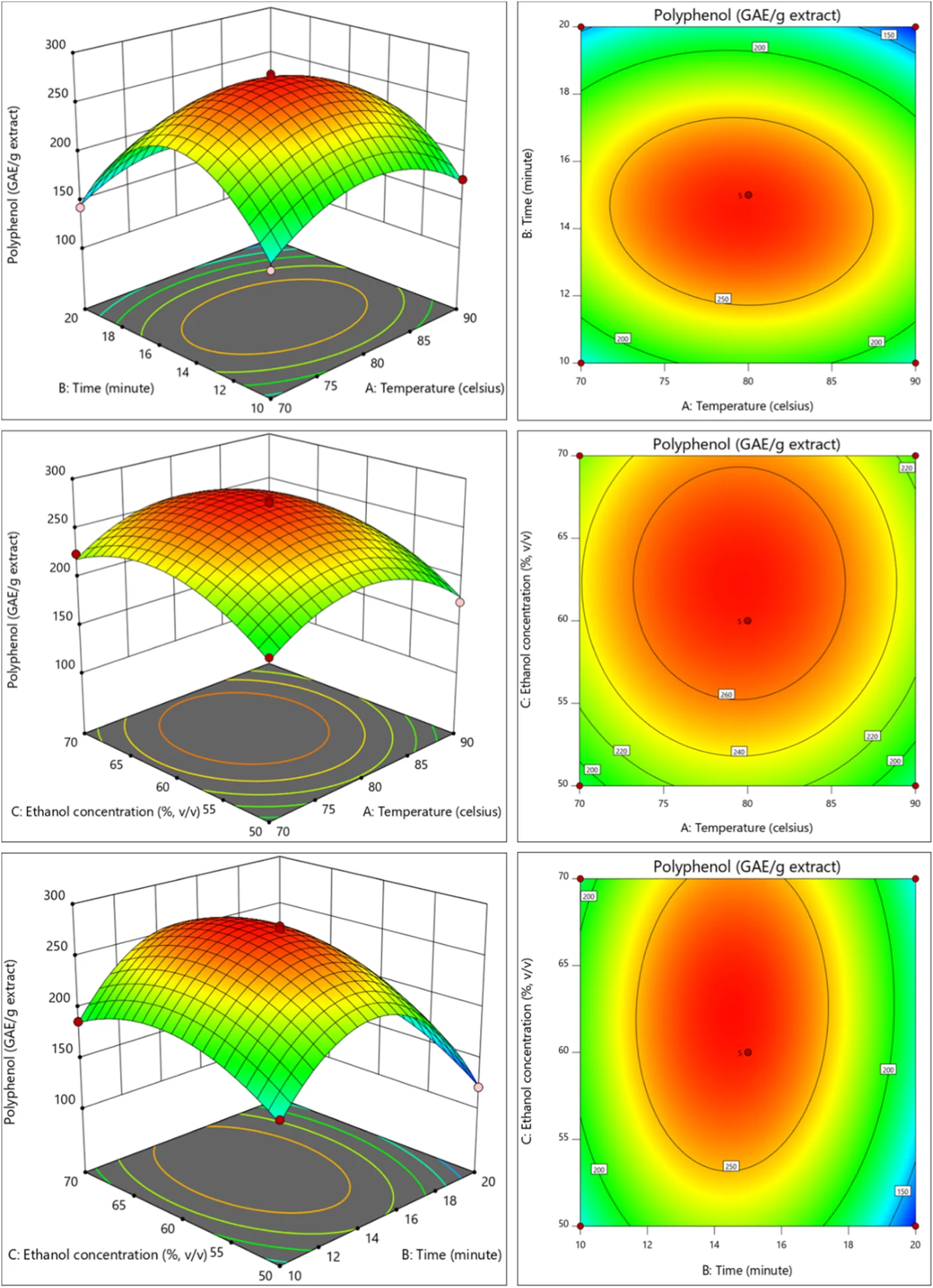
Response surface plots illustrating the interaction effects of extraction factors (temperature, time, ethanol concentration) on polyphenol yield (one factor held at center in each plot).

By analyzing the response surface and its derivatives, the model-predicted optimum conditions were determined to be an extraction temperature of 79.22 °C, an extraction time of 14.55 min, and an ethanol concentration of 60.42%, with the solid-to-liquid ratio fixed at 1:10 (w/v). For practical application, these conditions were rounded to 79 °C, 15 min, and 60% ethanol, respectively. Under the unrounded optimal conditions, the model predicted a maximum polyphenol content of 276.20 mg GAE/g extract (Fig. 4). An independent validation experiment conducted under the rounded optimal conditions yielded an experimental polyphenol content of 275.37 ± 1.55 mg GAE/g extract, showing good agreement with the model prediction. These results supported the adequacy of the response surface model for predicting polyphenol content within the investigated experimental domain. However, broader process robustness beyond the studied factor ranges would require additional validation under extended extraction conditions.

**Fig. 4:**
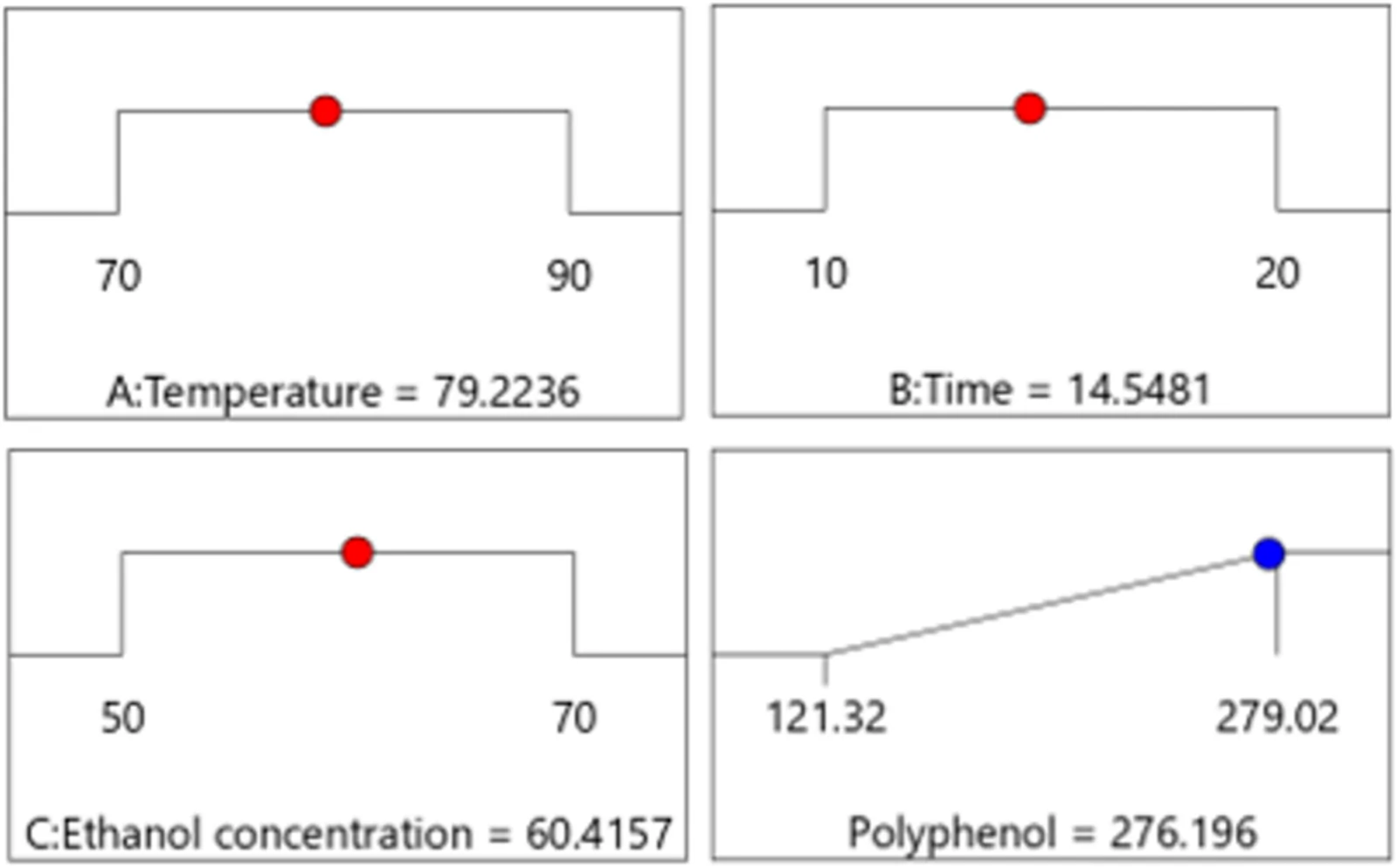
Predicted versus experimental polyphenol yields under various conditions, and the optimum extraction condition indicated by the model.

The use of ethanol–water (60%) as the extraction solvent not only maximized polyphenol yield but also reflects a sustainable and food-grade approach, which is advantageous for potential nutraceutical development. Compared with longer extraction times or higher solvent ratios reported in other plant matrices, the relatively short ultrasonication time (15 min) and moderate temperature (79 °C) employed here highlight the energy efficiency and scalability of this optimized process. Thus, the optimized ultrasound-assisted extraction is not only statistically valid but also practically suitable for translation into large-scale production of functional food ingredients. Interestingly, the optimized extraction conditions identified in the present study differed from those reported in our previous investigations on alkaloid-rich and flavonoid-rich extracts from *M. alliacea* flowers [12], [13]. In particular, the polyphenol-oriented extraction employed a lower ethanol concentration than the alkaloid-focused extraction, suggesting differences in solvent polarity requirements among phytochemical classes. Moreover, while previous studies emphasized antioxidant, antibacterial, and anti-obesity properties associated with alkaloid and flavonoid enrichment, the present work focused on anti-inflammatory and antidiabetic activities associated with polyphenol enrichment. These findings collectively indicate that different phytochemical fractions obtained from the same botanical source may exhibit distinct extraction behaviors and biological profiles. A recognized limitation of the present study is that the response variable was specifically defined as total polyphenol content per mass of dried extract (mg GAE/g extract) to target phytochemical enrichment rather than the absolute extraction recovery from the raw plant biomass. Consequently, because extraction yield (% w/w dry flower) was not co-optimized, these data denote polyphenol purity/concentration in the extract rather than total mass recovery. Although this approach was suitable for obtaining a polyphenol-rich extract for biological activity assessment, future studies should incorporate extraction recovery parameters to provide a more comprehensive evaluation of extraction efficiency.

Both the optimized extract (OMAE) and the unoptimized extract (UMAE) were subjected to UHPLC–MS profiling to compare their phytochemical compositions. The unoptimized extract (UMAE) was prepared under non-optimized baseline extraction conditions selected from preliminary experiments and conventional extraction parameters commonly reported for medicinal plant extraction. Therefore, UMAE was not intended to represent the poorest extraction condition, but rather to serve as a practical reference for evaluating the improvement achieved through statistical optimization. Subsequently, the extracts were evaluated in biological activity assays and benchmarked against standard reference compounds, as described in Sections 3.3, 3.4.

### 3.3 UHPLC–MS profiling of UMAE and OMAE extracts

UHPLC–MS analysis revealed that both OMAE and UMAE contained nine major chromatographic peaks (Fig. 5). Since the present study employed single-quadrupole LC–MS for preliminary phytochemical profiling, all compound assignments should be considered tentative. Comparative chromatographic analysis showed that the total peak area of OMAE was approximately 3.51-fold higher than that of UMAE (Table 3), suggesting that the optimization process mainly improved the relative abundance and concentration of detectable polyphenolic constituents rather than substantially changing the overall chromatographic pattern. Among the detected peaks, compounds 6 and 7 exhibited the most pronounced increases in relative abundance in OMAE and were therefore selected for further tentative characterization and discussion.

**Fig. 5:**
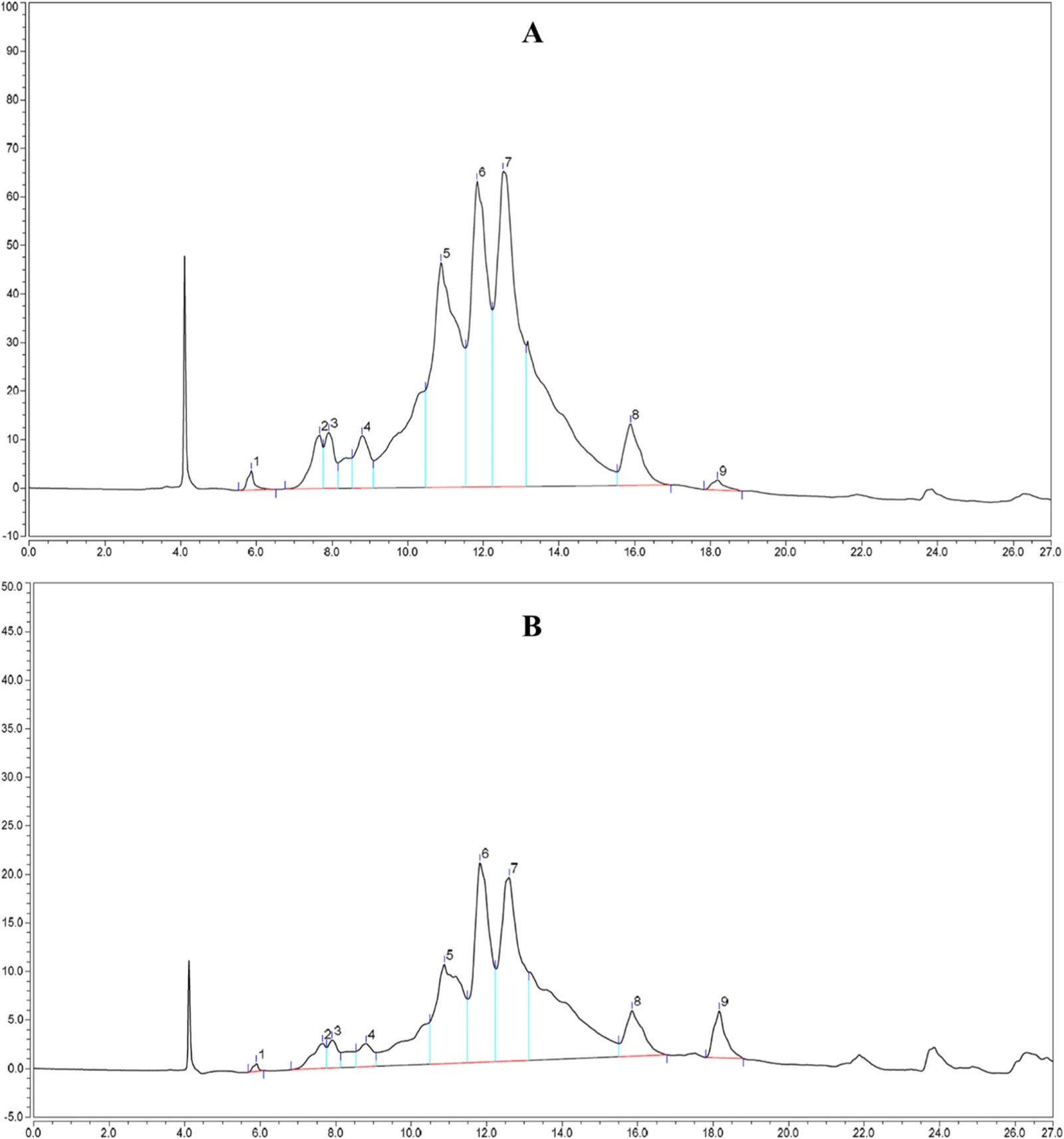
LC–MS chromatograms of *M. alliacea* extracts. (A) OMAE; (B) UMAE. Nine peaks (1–9) were detected in both extracts, with larger peak areas in OMAE. Peaks 6 and 7 were provisionally identified as luteolin-7-O-glucoside and luteolin-7-O-glucuronide, respectively.

**Table 3:** Comparative LC–MS peak data of optimized (OMAE) and unoptimized (UMAE) extracts.

Peak No.	Retention Time (min) OMAE/UMAE	Tentative Compound	Peak Area (OMAE)	Peak Area (UMAE)	Increase (↑) or decrease (↓) in change (times)
1	5.87/5.90	Not identified	0.827	0.123	↑, 6.72
2	7.67/7.65	Not identified	3.899	1.099	↑, 3.55
3	7.91/7.91	Not identified	3.471	0.867	↑, 4.00
4	8.79/8.80	Not identified	4.719	1.020	↑, 4.63
5	10.88/10.87	Not identified	35.897	7.857	↑, 4.57
6	11.83/11.82	Luteolin-7-O-glucoside	33.711	10.260	↑, 3.29
7	12.52/12.59	Luteolin-7-O-glucuronide	40.623	11.679	↑, 3.48
8	15.88/15.85	Not identified	6.613	2.550	↑, 2.59
9	18.18/18.16	Not identified	0.816	1.763	↓, 2.16
**Total area**	–	–	130.577	37.218	↑, 3.51

**Table 4:** IC_50_ (μg/mL) values obtained from various methods.

Method	Sample	Regression model	IC_50_ value (μg/mL)
**ABTS**^•^**^+^**	Vitamin C	y = 7.0914x + 0.1934 (R^2^ = 0.9989)	7.03 ± 0.19
	OMAE	y = 3.2749x – 1.3013 (R^2^ = 0.9960)	15.58 ± 0.33
	UMAE	y = 0.6708x + 0.4806 (R^2^ = 0.9996)	73.83 ± 0.86
**DPPH**	Vitamin C	y = 8.5963x – 2.0250 (R^2^ = 0.9983)	6.05 ± 0.04
	OMAE	y = 4.2397x – 0.3538 (R^2^ = 0.9985)	11.88 ± 0.28
	UMAE	y = 0.6901x + 0.2014 (R^2^ = 0.9969)	72.21 ± 2.32
**BSA**	Diclofenac	y = 1.2414x + 1.0292 (R^2^ = 0.9952)	39.45 ± 1.21
	OMAE	y = 1.2451x + 1.7476 (R^2^ = 0.9962)	38.77 ± 0.93
	UMAE	y = 0.3338x + 0.7305 (R^2^ = 0.9984)	145.69 ± 2.31
**RBCs**	Diclofenac	y = 1.7456x + 0.3120 (R^2^ = 0.9976)	28.47 ± 0.68
	OMAE	y = 1.6127x + 0.4201 (R^2^ = 0.9988)	30.74 ± 0.18
	UMAE	y = 0.3454x – 0.0181 (R^2^ = 0.9983)	144.88 ± 3.01
***α*-Amylase**	Acarbose	y = 3.0574x + 2.9596 (R^2^ = 0.9912)	15.39 ± 0.30
	OMAE	y = 1.4920x + 3.8589 (R^2^ = 0.9864)	30.94 ± 0.54
	UMAE	y = 0.3389x + 2.3628 (R^2^ = 0.9947)	140.58 ± 0.57
***α*-Glucosidase**	Acarbose	y = 6.7196x – 1.7859 (R^2^ = 0.9970)	7.71 ± 0.19
	OMAE	y = 3.8286x – 0.9849 (R^2^ = 0.9982)	13.32 ± 0.19
	UMAE	y = 0.7470x – 0.4690 (R^2^ = 0.9998)	67.57 ± 1.49

Specifically, based on UHPLC-MS qualitative profiling of UMAE (S Figs. 1 and 2), compound 6 exhibited a deprotonated molecular ion [M − H]^-^ at *m*/*z* 447.62 and an in-source fragment ion at *m*/*z* 285.49 (S Fig. 1), characteristic of the cleavage of a hexose moiety from a flavonoid O-hexoside. In addition, the UV-Vis spectrum of compound 6 showed three absorption maxima at 241.72, 282.42, and 335.34 nm (S Fig. 2), with band I at 335.34 nm being typical of the flavone backbone, particularly luteolin (*λ*_max_ = 333 nm) [25]. This value is significantly lower than the *λ*_max_ reported for flavonols such as kaempferol (about 360 nm) [26] or quercetin (about 370 nm) [27]. Together with a retention time of 11.82 min (Fig. 5B), MS data (*m*/*z* 447.62 → 285.49) (S Fig. 1), and UV spectral characteristics (S Fig. 2), these findings tentatively suggest that compound 6 may correspond to a luteolin O-hexoside derivative, most likely luteolin-7-O-glucoside. The theoretical monoisotopic mass of the predicted compound (luteolin-7-O-glucoside, C_21_H_20_O_11_, 448.10 Da) include: *m*/*z* [M − H]^-^ calculated = 447.09 and *m*/*z* of the aglycone fragment = 285.05. Taken together, these analyses indicate that compound 6 (Fig. 6) in the present study closely matches previous reports describing UV data and LC-MS features of luteolin-7-O-glucoside (cynaroside) in medicinal plants [28], [29], [30].

**Fig. 6:**
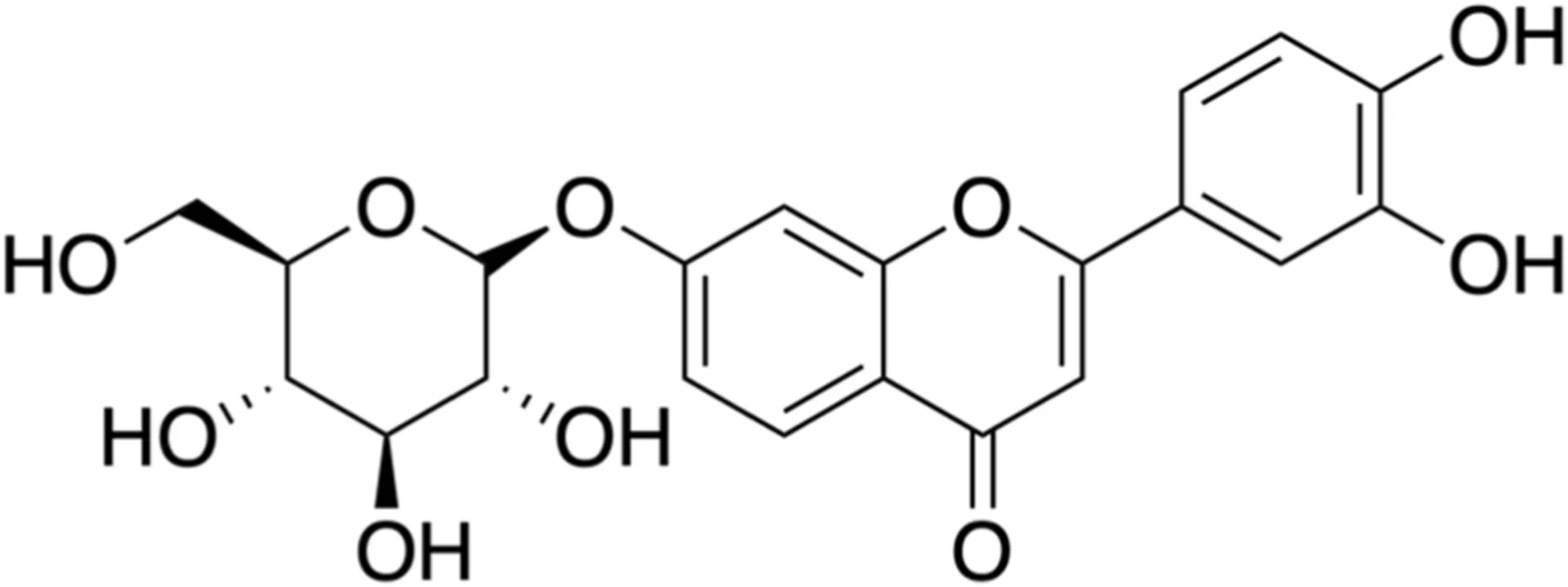
Compound 6 (luteolin-7-O-glucoside).

The peak area of compound 6 in OMAE increased 3.29-fold compared with UMAE, suggesting that the optimization process not only enhanced total polyphenol yield but also preferentially enriched water-soluble glycosides. The putatively assigned luteolin-7-O-glucoside has been reported to exert antioxidant and anti-inflammatory effects by inhibiting NF-κB, MAPK, and STAT3 signaling pathways, reducing the production of pro-inflammatory cytokines, and upregulating antioxidant enzymes such as HO-1 and Nrf2 [31], [32], [33]. This putative compound has also been reported to prevent protein denaturation associated with inflammation and protect cells from oxidative stress [31], [34]. Moreover, the putatively assigned luteolin-7-O-glucoside has been reported to inhibit α-amylase and α-glucosidase activities, thereby contributing to glycemic control, although its potency is generally lower than that of free luteolin [33]. Overall, the tentative presence of luteolin-7-O-glucoside in OMAE may partially contribute to the observed antioxidant, anti-inflammatory, and enzyme inhibitory activities. The enrichment of luteolin glycosides in the optimized extract is consistent with our previous findings demonstrating that *M. alliacea* flowers contain abundant flavonoid-related constituents and phenolic derivatives [13]. However, further confirmation using MS/MS analysis, authentic standards, and bioactivity-guided studies is required.

Similarly, based on UHPLC-MS qualitative profiling of the non-optimized extract (S Figs. 3 and 4), compound 7 exhibited a deprotonated molecular ion [M − H]^-^ at *m*/*z* 461.44 and an in-source fragment ion at *m*/*z* 285.15 (S Fig. 3), indicative of the loss of a glucuronic acid moiety (about 176 Da) yielding the luteolin aglycone [35], [36]. In addition, the UV–Vis spectrum of compound 7 showed three absorption maxima at 242.55, 281.06, and 333.13 nm (S Fig. 4), with band I falling within the range typical for the flavone skeleton, consistent with luteolin, similar to compound 6 [25].

Taking into account the retention time of compound 7 (12.59 min, Fig. 5B), the measured MS data (*m*/*z* 461.44 → 285.15) (S Fig. 3), and UV spectral features (S Fig. 4), this compound is most likely a luteolin O-glucuronide derivative, specifically luteolin-7-O-glucuronide. The theoretical monoisotopic mass of the predicted compound (luteolin-7-O-glucuronide, C_21_H_18_O_12_, 462.08 Da) include: *m*/*z* [M − H]^-^ calculated = 461.07 and aglycone fragment calculated = 285.05. Collectively, these results demonstrate that compound 7 (Fig. 7) in this study closely resembles previous reports describing UV and LC-MS data for luteolin-7-O-glucuronide in medicinal plants [31], [37].

**Fig. 7:**
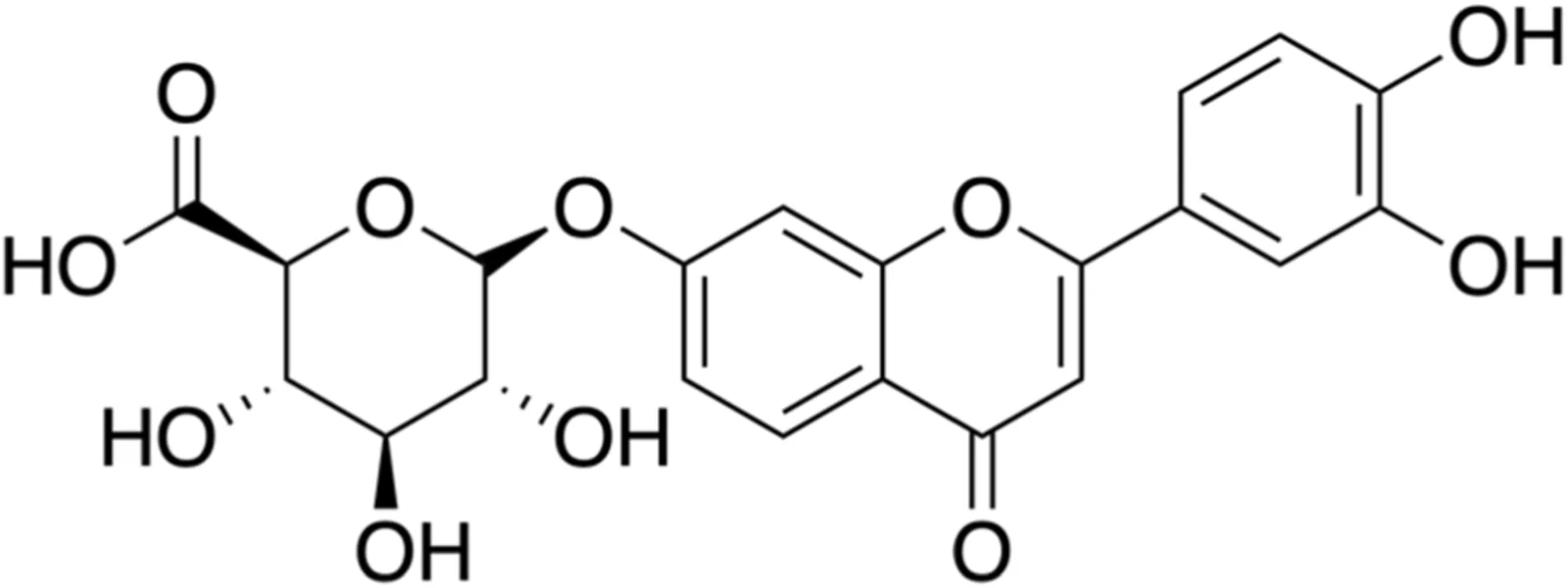
Compound 7 **(**luteolin-7-O-glucuronide**)**.

The putatively assigned luteolin-7-O-glucuronide is reported to be a glucuronic acid conjugate commonly formed during flavonoid metabolism, enhancing aqueous solubility and facilitating excretion while retaining the biological activity of luteolin aglycone. Notably, the peak area of compound 7 in OMAE increased 3.48-fold compared with UMAE. Importantly, pharmacokinetic studies have indicated that putative luteolin-7-O-glucuronide can be hydrolyzed back into luteolin at target tissues by β-glucuronidase, thereby exerting antioxidant, anti-inflammatory, and hypoglycemic effects [38], [39], [40], [41]. Thus, the enrichment of peak 7 in OMAE may be associated with the enhanced biological activities observed in the optimized extract, although its precise contribution remains to be experimentally verified.

The concurrent enrichment of the putatively assigned luteolin-7-O-glucoside (peak 6) and luteolin-7-O-glucuronide (peak 7) indicates that OMAE not only enhances the pool of potentially bioactive luteolin aglycone but also enriches its glycosylated and glucuronidated derivatives, which are of critical importance in pharmacokinetics. Glycosides improve solubility and intestinal absorption, whereas glucuronides act as storage or transport forms that can be hydrolyzed to release active luteolin at inflamed tissues or under oxidative stress conditions. The concurrent enrichment of the provisionally assigned luteolin-7-O-glucoside (peak 6) and luteolin-7-O-glucuronide (peak 7) is statistically associated with the improved *in vitro* bioactivity profiles of OMAE relative to UMAE. However, this association should strictly not be interpreted as demonstrated causality. Because single-quadrupole LC–MS was utilized and seven of the nine detected chromatographic peaks remain unidentified, and because pure-compound isolation or bioactivity-guided fractionation was not performed, attributing the observed antioxidant, membrane-stabilizing, or enzyme-inhibiting effects specifically to these two tentative flavonoids would be premature. Instead, the enhanced bioactivities are more plausibly the net outcome of synergistic or additive interactions among the broader complex matrix of co-extracted polyphenols. These qualitative findings serve as preliminary chromatographic characterization and highlight promising candidate molecules for future bioactivity-guided validation. However, additional studies involving advanced structural characterization and bioactivity-guided fractionation are necessary before definitive conclusions regarding active constituents can be established.

Although the single-quadrupole MS employed in this study does not permit absolute quantification, comparative analysis of peak intensities offers reliable evidence of relative enrichment. Nevertheless, the phytochemical characterization in the present study remains preliminary. Although nine major chromatographic peaks were detected, only two compounds could be tentatively assigned based on UV and single-quadrupole MS data. Consequently, direct correlations between specific constituents and the observed biological activities should be interpreted cautiously. More comprehensive characterization using high-resolution LC–MS/MS, authentic reference standards, and quantitative analysis of major constituents will be necessary in future studies to confirm compound identities and better elucidate the chemical basis of the extract's bioactivities. These qualitative LC–MS observations are consistent with the quantitative Folin–Ciocalteu assay, which showed significantly higher total polyphenol content in OMAE (275.37 ± 1.55 mg GAE/g extract) than in UMAE. Taken together, the LC-MS profiling and TPC data suggest that optimized ultrasound-assisted extraction increased overall polyphenol yield while enhancing the relative abundance of putative phenolic constituents, thereby supporting the improved biological potential of the extract.

Although the tentative enrichment of luteolin glycosides and glucuronides in OMAE is chemically interesting, the present findings remain preliminary because compound identification was based on single-quadrupole LC–MS analysis. Therefore, additional studies using high-resolution MS/MS, authentic reference standards, and *in vivo* models are necessary to validate the contribution of these compounds to the observed biological activities and to assess their translational relevance. While the current data provide preliminary chemical evidence supporting the enhanced bioactivity of OMAE, *in vivo* studies using relevant disease models (e.g., inflammation, metabolic disorders, or oxidative stress–related conditions) are needed to confirm the bioavailability and pharmacodynamic advantages of OMAE. Moreover, pharmacokinetic profiling could clarify how the interplay between aglycone luteolin, its glycosylated derivatives, and glucuronide conjugates contributes to tissue distribution and sustained activity. Such investigations would not only strengthen the mechanistic understanding of OMAE's bioactivity but also accelerate its potential development as a standardized functional extract or phytopharmaceutical candidate.

### 3.4 Biological activities of UMAE and OMAE extracts

#### 3.4.1 In vitro antioxidant activity

The antioxidant activity of OMAE and UMAE was examined via ABTS^•+^ and DPPH radical scavenging assays, using ascorbic acid as a positive control. Both extracts exhibited concentration-dependent radical neutralization, with OMAE demonstrating a noticeably higher efficacy than UMAE (Fig. 8). As expected, ascorbic acid (a potent antioxidant) showed the strongest activity overall, achieving 6.23 ± 0.77% ABTS^•+^ scavenging at 1 μg/mL and 71.38 ± 1.97% at 10 μg/mL; similarly, it scavenged 5.92 ± 0.88% of DPPH at 1 μg/mL and 84.40 ± 1.60% at 10 μg/mL.

**Fig. 8:**
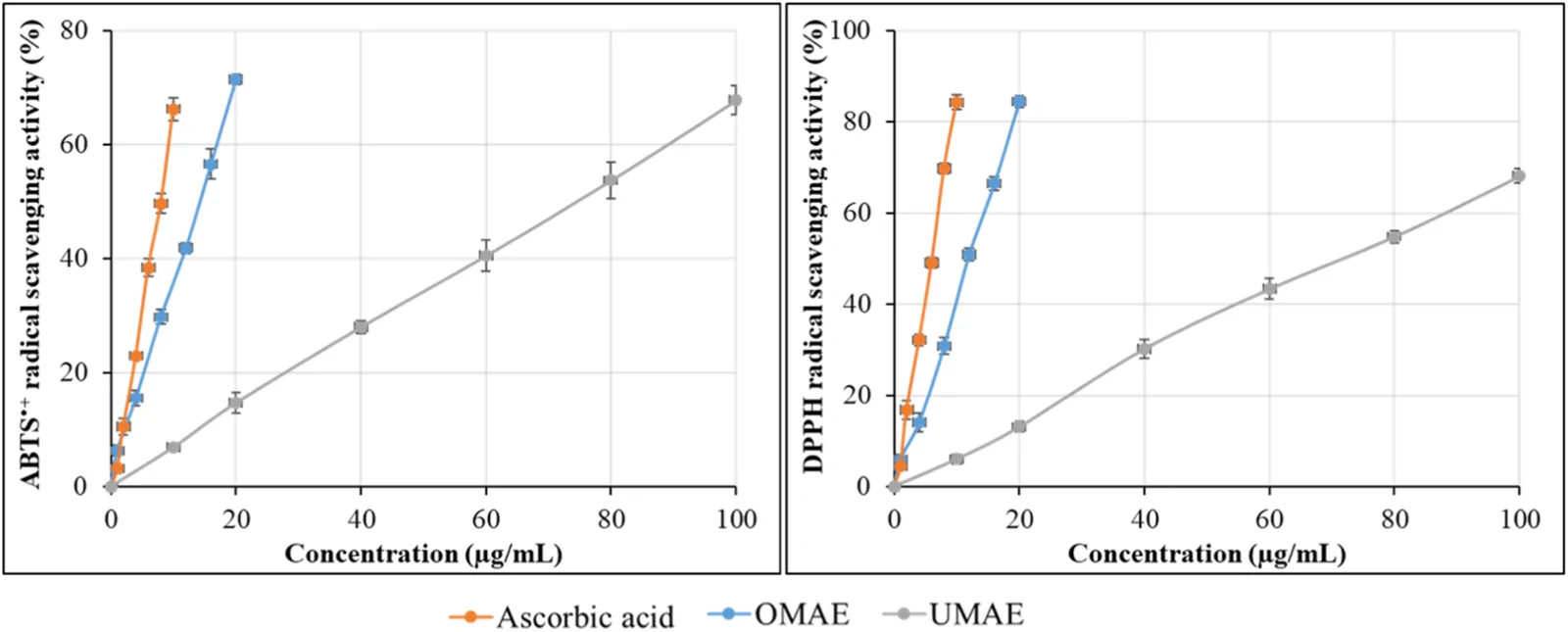
Concentration-dependent ABTS^•+^ and DPPH free radical scavenging by ascorbic acid (standard), optimized *M. alliacea* extract (OMAE), and unoptimized extract (UMAE). Data are presented as mean ± SD of three independent biological replicates (n = 3), with each sample measured across three technical replicates. Error bars represent standard deviations.

OMAE was quite effective even at low concentrations. At 1 μg/mL, OMAE quenched 3.14 ± 0.91% of ABTS^•+^ radicals, and this increased steadily to 66.16 ± 0.99% at 20 μg/mL. A similar trend was observed for DPPH: OMAE caused 4.49 ± 1.15% DPPH inhibition at 1 μg/mL, reaching 84.15 ± 1.26% inhibition at 20 μg/mL. In contrast, the UMAE required higher concentrations to achieve comparable effects. Over the range of 10 to 100 μg/mL, UMAE scavenged 5.98 ± 1.03% to 68.06 ± 1.58% of ABTS^•+^, and showed a DPPH scavenging range of 9.42 ± 2.18% to 71.71 ± 1.63%, indicating that OMAE is more potent. These results are graphically depicted in Fig. 8.

The superior radical-scavenging ability of OMAE can be attributed to its higher concentration of polyphenolic constituents. Free radical-induced oxidative stress is implicated in many chronic conditions such as diabetes, arthritis, cancer, cardiovascular diseases, and aging. Indeed, elevated levels of reactive oxygen species have been observed in diabetic patients [42]. Polyphenols act as antioxidants by donating electrons or hydrogen atoms to neutralize free radicals, or by chelating metal ions, thereby stabilizing reactive species and rendering them non-toxic to biomolecules [42]. The strong radical-scavenging activity of the polyphenol-rich *M. alliacea* extract provides preliminary *in vitro* evidence of antioxidant potential. Since oxidative stress is associated with several chronic disorders, including diabetes, these findings suggest that the extract may warrant further investigation in more biologically relevant models. However, the present assays do not directly demonstrate therapeutic efficacy *in vivo*.

#### 3.4.2 In vitro anti-inflammatory activity

We assessed the anti-inflammatory effects of the extracts by their ability to inhibit protein denaturation and to protect erythrocyte membranes from lysis (membrane stabilization). Protein denaturation is a common pathway in inflammation, and inhibiting protein denaturation is one mechanism by which anti-inflammatory agents can exert their effects [43]. Additionally, since lysosomal enzymes released during inflammation can damage tissues, stabilizing membranes (such as RBC membranes, which are analogous to lysosomal membranes) is another indicator of anti-inflammatory potential [44].

Both OMAE and UMAE inhibited heat-induced BSA denaturation in a dose-dependent manner, as did the reference drug diclofenac (Fig. 9). OMAE showed notable efficacy, with inhibition increasing from 2.92 ± 0.59% at the lowest tested concentration (1.56 μg/mL) to 62.70 ± 2.12% at 50 μg/mL. Across the tested concentration range, no statistically significant differences in protein denaturation inhibition were observed between OMAE and diclofenac (p > 0.05, one-way ANOVA followed by Tukey's test). At 50 μg/mL, the inhibitory activity of OMAE was also not significantly different from that of diclofenac under the conditions of the present assay. However, the lack of a statistically significant difference should not be interpreted as evidence of pharmacological or therapeutic equivalence between a multicomponent botanical extract and a single-molecule reference drug. UMAE also inhibited protein denaturation, but to a much lesser extent (IC_50_ = 145.69 ± 2.31 μg/mL). This indicates that optimizing the extraction greatly enhanced the anti-denaturation activity, likely due to the enriched polyphenol content in OMAE.

**Fig. 9:**
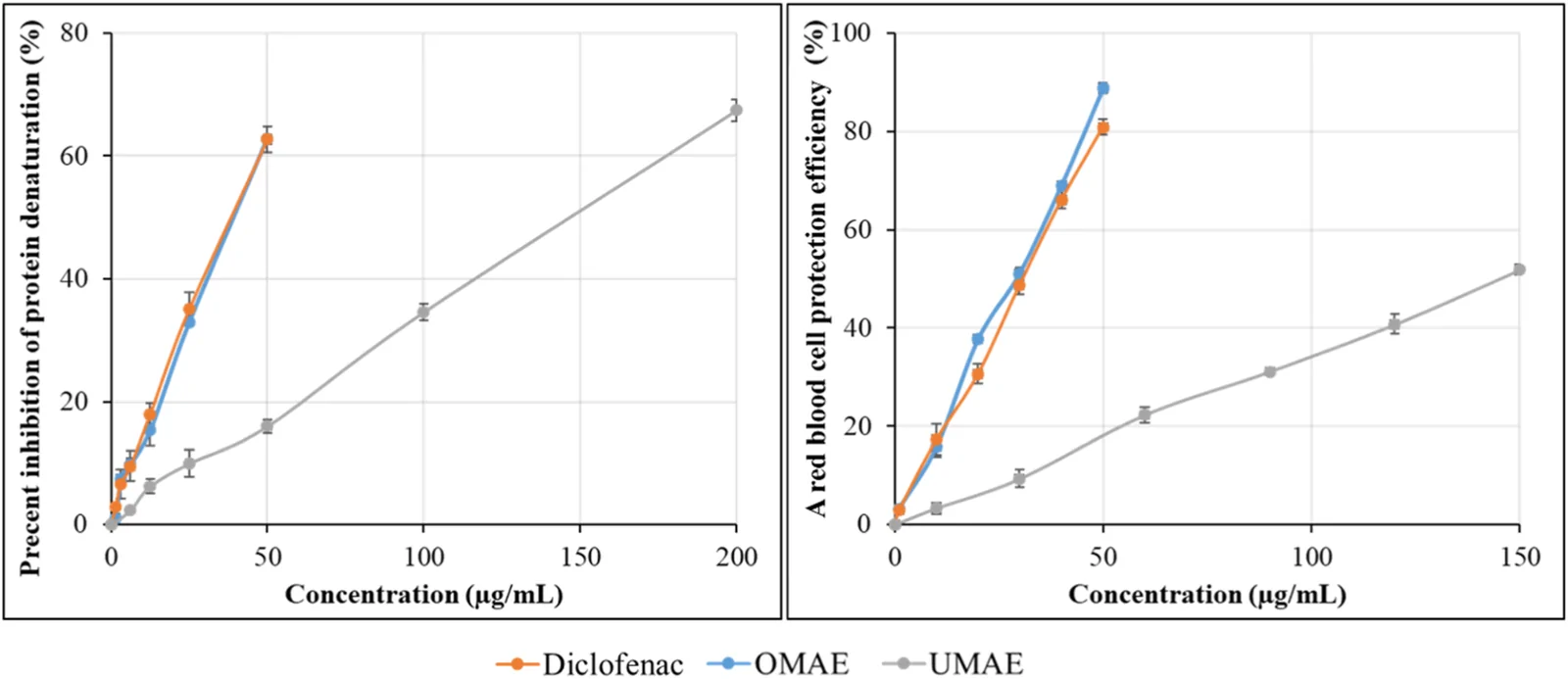
Inhibition of protein denaturation (BSA assay) and RBC hemolysis by diclofenac, OMAE, and UMAE as a function of concentration. Data are presented as mean ± SD of three independent biological replicates (n = 3), with each measurement performed in technical triplicates. Error bars denote the standard deviation of biological replicates.

In the erythrocyte protection assay, OMAE again demonstrated superior activity over UMAE (Fig. 9). At 10 μg/mL, OMAE protected 17.31 ± 2.05% of RBCs from hemolysis, which was about 5.17 times the protection afforded by UMAE (3.35 ± 1.17%). As the concentration of OMAE increased, RBC protection rose dramatically, from 2.88 ± 0.86% at 1 μg/mL to 80.85 ± 1.03% at 50 μg/mL. At comparable concentrations, OMAE showed no statistically significant difference from diclofenac in membrane stabilization activity under the conditions of the present *in vitro* assay (p > 0.05, one-way ANOVA followed by Tukey's test). These findings indicate that OMAE exhibited measurable membrane-stabilizing activity and may contain bioactive constituents capable of reducing erythrocyte lysis under thermal stress. However, these *in vitro* findings should not be interpreted as evidence of bioequivalence or clinical equivalence with diclofenac, as formal equivalence testing, pharmacokinetic assessments, and *in vivo* efficacy studies were not performed.

Polyphenols are well-known to contribute to anti-inflammatory effects, partly through their antioxidant action. By scavenging reactive species and preventing protein modifications, they can interrupt the inflammatory cascade [45]. Our findings show that the polyphenol-rich *M. alliacea* extract (OMAE) can effectively inhibit processes (protein denaturation, membrane lysis) that are central to inflammation. Thus, OMAE demonstrated activity in multiple *in vitro* models commonly associated with anti-inflammatory potential, although further mechanistic and *in vivo* studies are required to confirm these observations. The calculated IC_50_ values for these anti-inflammatory assays are discussed in Section 3.4.4.

#### 3.4.3 In vitro carbohydrate-hydrolyzing enzyme inhibitory activity

Type 2 diabetes mellitus is characterized by impaired insulin action and elevated postprandial blood glucose levels. One therapeutic approach to manage blood glucose is to inhibit carbohydrate-digesting enzymes in the digestive tract, *α*-amylase (which breaks down starches) and *α*-glucosidase (which breaks down disaccharides). Inhibiting these enzymes slows the release of glucose from complex carbohydrates, thereby flattening postprandial glucose spikes [46]. Acarbose, a pharmaceutical *α*-glucosidase inhibitor, is widely used for this purpose, although it can cause gastrointestinal side effects (such as bloating, soft stools) with long-term use [46], [47].

In this study, both OMAE and UMAE were tested for their ability to inhibit *α*-amylase and *α*-glucosidase, and their efficacy was compared with that of acarbose. The results showed clear differences between the optimized and unoptimized extracts. OMAE exhibited considerable inhibitory effects on both enzymes, whereas UMAE was much less potent (Fig. 10). Specifically, for *α*-amylase, OMAE at concentrations from 1.56 to 50 μg/mL achieved 4.02 ± 0.89% to 76.40 ± 3.00% inhibition. At the highest tested concentration (50 μg/mL), OMAE's *α*-amylase inhibition was about 3.45-fold greater than that of UMAE at the same concentration. Likewise, OMAE effectively inhibited *α*-glucosidase, with 3.72 ± 0.34% inhibition at 1 μg/mL and 75.78 ± 1.59% at 20 μg/mL. At 20 μg/mL, OMAE was 5.34 times more potent against *α*-glucosidase than UMAE. These enhancements reflect the higher polyphenol content in OMAE, as many polyphenolic compounds are known to interfere with these digestive enzymes.

**Fig. 10:**
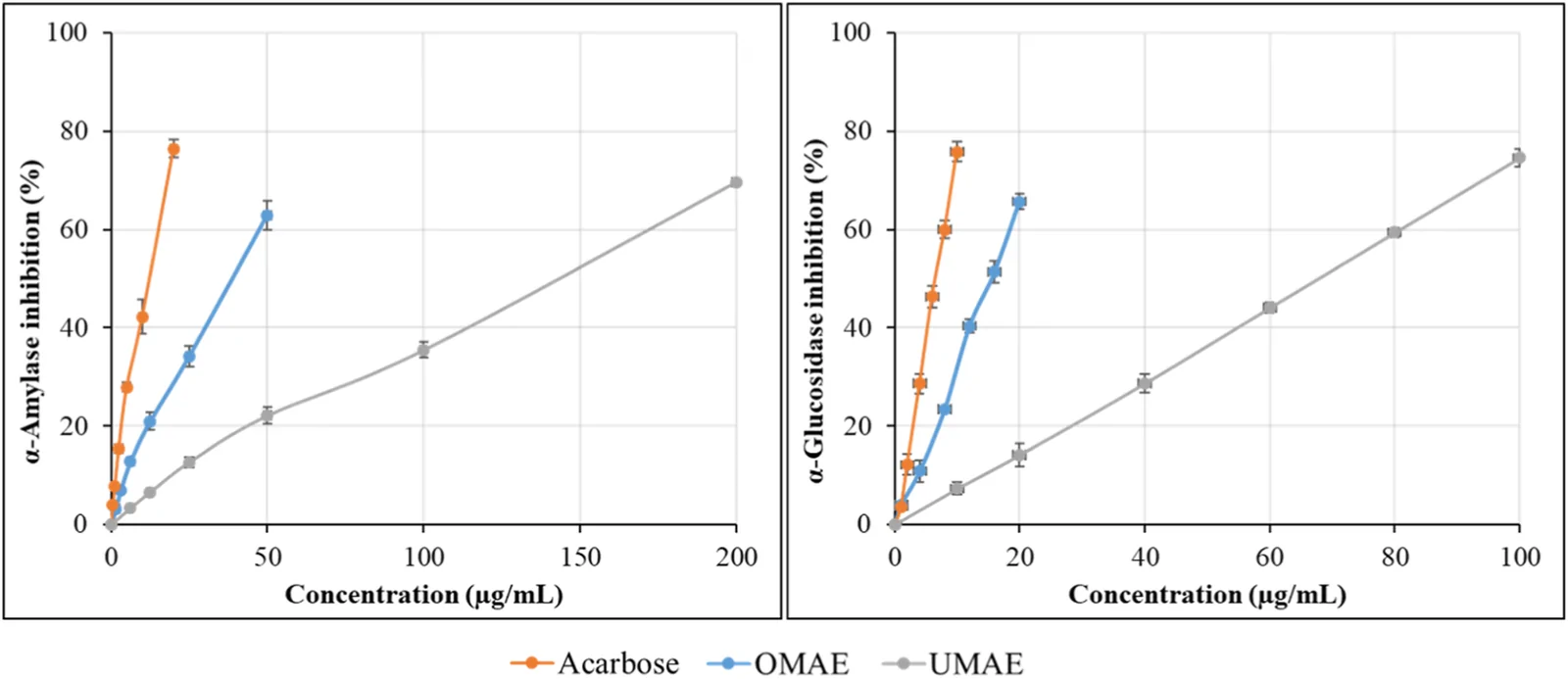
Inhibition of *α*-amylase and *α*-glucosidase enzymes by acarbose (standard antidiabetic), OMAE, and UMAE (percentage inhibition versus inhibitor concentration). Data are presented as mean ± SD of three independent biological replicates (n = 3), with each measurement performed in technical triplicates. Error bars denote the standard deviation of biological replicates.

Although OMAE significantly outperformed UMAE, acarbose remained the most potent inhibitor in absolute terms. In our assays, acarbose exhibited IC_50_ values of 15.39 ± 0.30 μg/mL for *α*-amylase and 7.71 ± 0.19 μg/mL for *α*-glucosidase, confirming its strong inhibitory activity. OMAE's IC_50_ values were higher (less potent) at 30.94 ± 0.54 μg/mL for *α*-amylase and 13.32 ± 0.19 μg/mL for *α*-glucosidase (Section 3.4.4). In other words, OMAE was about 2 times less active than acarbose on *α*-amylase and about 1.7 times less on *α*-glucosidase. Meanwhile, UMAE was far weaker (IC_50_ about 140.6 μg/mL for *α*-amylase and 67.6 μg/mL for *α*-glucosidase), indicating minimal enzyme inhibition capacity unless used at very high concentrations.

The ability of plant extracts to inhibit *α*-amylase and *α*-glucosidase is largely attributed to their polyphenolic content [48]. Polyphenols, especially those with multiple hydroxyl groups, can bind to the active sites or allosteric sites of these enzymes through hydrogen bonding and hydrophobic interactions, thus blocking the access of carbohydrate substrates [48]. The much stronger enzyme inhibition observed with OMAE correlates with its greater polyphenol concentration relative to UMAE. These findings indicate that the optimized extract exhibited potent *in vitro* inhibitory activity against alpha-amylase and alpha-glucosidase, suggesting its potential to modulate carbohydrate digestion under the tested conditions. However, the term “antidiabetic efficacy” is deliberately avoided because inhibition of digestive enzymes *in vitro* reflects biochemical activity rather than regulation of physiological glycemic homeostasis. Demonstration of *in vivo* efficacy would require consideration of factors such as gastrointestinal stability, intestinal absorption, bioavailability, metabolism, and systemic pharmacodynamics, which were beyond the scope of the present *in vitro* study. Although OMAE was less potent than acarbose under the present experimental conditions, its polyphenol-rich composition may justify further investigation as a source of bioactive compounds with enzyme inhibitory properties.

#### 3.4.4 Bioactivity comparisons in terms of IC_50_

To quantitatively compare the strength of antioxidant, anti-inflammatory, and antidiabetic activities, IC_50_ values were determined for OMAE, UMAE, and the standard references. The IC_50_ is the concentration required to achieve 50% of the maximal effect and is a convenient metric: lower IC_50_ values denote higher potency. An agent is classified as a very strong antioxidant if its IC_50_ is below 50 μg/mL, strong from 50 to 100 μg/mL, moderate from 101 to 150 μg/mL, and weak if above 150 μg/mL [49].

As shown in Table 4, OMAE had IC_50_ values of 15.58 ± 0.33 μg/mL (ABTS assay) and 11.88 ± 0.28 μg/mL (DPPH assay). These values classify OMAE as a “very strong” antioxidant and indicate substantial *in vitro* radical-scavenging activity. Although the IC_50_ values were numerically close to those of ascorbic acid, this comparison should be interpreted with caution because OMAE is a crude botanical extract whereas ascorbic acid is a purified reference compound. These data confirm that the optimization process markedly improved the extract's antioxidant efficacy, consistent with the higher polyphenol content in OMAE. Numerous studies support the direct correlation between polyphenol levels and antioxidant power, as polyphenols can readily donate electrons to neutralize free radicals [42]. Thus, the higher radical scavenging capacity of OMAE aligns with its enriched polyphenolic profile.

The IC_50_ values for inhibition of protein denaturation (BSA assay) and RBC hemolysis are also summarized in Table 4. OMAE inhibited protein denaturation with IC_50_ = 38.77 ± 0.93 μg/mL and protected RBC membranes with IC_50_ = 30.74 ± 0.18 μg/mL. These IC_50_ values were numerically comparable to those observed for diclofenac, with IC_50_ values of 39.45 ± 1.21 μg/mL for protein denaturation and 28.47 ± 0.68 μg/mL for RBC protection. However, it should be emphasized that the absence of statistically significant differences (p > 0.05) in these screening models only indicates that the responses did not differ significantly under the specific experimental conditions employed. It should not be interpreted as evidence of pharmacological equivalence or non-inferiority, particularly in the absence of a prespecified and appropriately powered non-inferiority analysis. Therefore, the comparison with diclofenac is presented solely as a reference benchmark to contextualize the observed *in vitro* inhibitory activity of the enriched extract. By contrast, UMAE was much less effective, with IC_50_ values of 145.69 ± 2.31 μg/mL (BSA denaturation) and 144.88 ± 3.01 μg/mL (RBC assay), which are roughly 3.8–4.7 times higher than those of OMAE. These results highlight that the optimized extract's richer polyphenol content translates into significantly greater anti-inflammatory action. Polyphenolic secondary metabolites often exert anti-inflammatory effects indirectly through their antioxidant activity (preventing oxidative damage that leads to inflammation) and directly by modulating inflammatory pathways [45]. The notable performance of OMAE in these assays supports the potential relevance of *M. alliacea* as a source of bioactive polyphenols for further investigation.

Acarbose, the positive control, had IC_50_ = 15.39 ± 0.30 μg/mL for *α*-amylase and 7.71 ± 0.19 μg/mL for *α*-glucosidase in our experiments, consistent with its high potency as an enzyme inhibitor [46]. OMAE showed IC_50_ values of 30.94 ± 0.54 μg/mL (*α*-amylase) and 13.32 ± 0.19 μg/mL (*α*-glucosidase). Thus, OMAE was about half as potent as acarbose against *α*-amylase (approximately 2.0-fold higher IC_50_) and about 1.7-fold less potent against *α*-glucosidase. Still, given that UMAE's IC_50_ values were 140.58 ± 0.57 μg/mL and 67.57 ± 1.49 μg/mL for *α*-amylase and *α*-glucosidase respectively, OMAE's inhibitory strength was improved by roughly 4.5–5.1 times over the unoptimized extract. While acarbose remains more powerful, OMAE exhibited notable enzyme inhibitory activity *in vitro* compared with the unoptimized extract, although its biological relevance requires further validation in cellular and *in vivo* models. Because OMAE is a crude botanical extract containing multiple phytochemical constituents whereas acarbose is a purified pharmaceutical compound, the observed differences in IC_50_ values should not be interpreted as evidence of equivalent therapeutic efficacy. Rather, these comparisons provide a useful benchmark for assessing the relative *in vitro* enzyme inhibitory potential of the optimized extract. Although OMAE was less potent than acarbose in these assays, its complex phytochemical composition may warrant further investigation of potential interactions among its constituents. The mechanism of enzyme inhibition by polyphenol-rich extracts is attributed to the binding of polyphenolic compounds to the enzymes, as discussed earlier [48]. Our data clearly show that the enhancements in polyphenol content achieved through extraction optimization have led to parallel improvements in biological activity across the board.

During the literature review and our own experiments, we found that classifying the strength of biological activities based on IC_50_ values is essential for an objective comparison between extracts and reference standards. Although no universal cut-off exists, many studies have proposed practical thresholds for different activities. For antioxidant assays, extracts with IC_50_ < 50 μg/mL are generally considered “very strong,” 50–100 μg/mL as “strong,” 101–150 μg/mL as “moderate,” and >150 μg/mL as “weak”. For anti-inflammatory *in vitro* models such as protein denaturation inhibition or RBC membrane stabilization, we propose that extracts with IC_50_ values comparable to or not exceeding 1.5-fold those of the standard drug (e.g., diclofenac) can be regarded as “strong”; 2–4 times higher as “moderate”; and >4 times higher as “weak.” Similarly, in α-amylase and α-glucosidase inhibition assays, extracts with IC_50_ values close to those of acarbose (<2-fold difference) may be considered “strong,” those 2–5 times higher as “moderate,” and >5 times higher as “weak.” This classification approach, informed by both previous studies and our present experimental findings, provides a useful framework to interpret the relative potencies of different extracts. Establishing such criteria not only facilitates comparative evaluation but also helps prioritize promising candidates for *in vivo* validation and future applications.

Despite these promising findings, several limitations must be acknowledged. First, the current study was restricted to *in vitro* assays; *in vivo* validation in animal models of oxidative stress, inflammation, and diabetes is necessary to confirm physiological relevance. Second, the use of a single-quadrupole MS limited compound identification, leaving seven major peaks uncharacterized. Therefore, the proposed associations between the putative luteolin derivatives and the observed biological activities should be considered preliminary and interpreted with caution. Third, the bioavailability of polyphenols, including putative luteolin derivatives, is generally low, which may attenuate their efficacy *in vivo*. Future work should therefore focus on *in vivo* pharmacological evaluation, advanced chemical profiling with MS/MS or NMR, and formulation strategies such as nanoencapsulation to enhance bioavailability. Therefore, the present findings should be interpreted as preliminary exploratory evidence rather than direct proof of therapeutic efficacy.

## 4 Conclusion

This study demonstrated that ultrasound-assisted extraction was successfully optimized to increase polyphenol concentration and enrichment in *Mansoa alliacea* flower extract, achieving a total polyphenol content of 275.37 ± 1.55 mg GAE/g extract under the selected conditions of 60% ethanol, 79 °C, 15 min, and a 1:10 (w/v) solid-to-solvent ratio. It should be emphasized that the response variable targeted polyphenol enrichment within the dry extract matrix rather than the cumulative mass of polyphenols recovered from the raw floral biomass. Therefore, further evaluations of crude extraction yield, total polyphenol recovery, and process economics are warranted to assess the scalability and practical feasibility of the optimized process.

Qualitative UHPLC–MS profiling revealed nine major chromatographic peaks, of which two were provisionally assigned as luteolin-7-O-glucoside and luteolin-7-O-glucuronide based on their UV spectra and single-quadrupole mass spectrometric characteristics. Both provisionally assigned constituents showed increased relative abundance in the optimized extract. The optimized extract also exhibited marked *in vitro* free-radical scavenging activity, inhibition of heat-induced protein denaturation and erythrocyte membrane lysis, and inhibition of the carbohydrate-hydrolyzing enzymes alpha-amylase and alpha-glucosidase compared with the unoptimized baseline extract.

However, these biological findings represent *in vitro* screening activities and should not be interpreted as evidence of *in vivo* anti-inflammatory or antidiabetic therapeutic efficacy. Furthermore, because seven of the nine major chromatographic peaks remain uncharacterized and bioactivity-guided isolation was not performed, any association between the provisionally assigned luteolin conjugates and the observed bioactivities remains correlative rather than demonstrating causality. The observed activities may reflect the combined effects and potential interactions among multiple constituents within the polyphenolic extract matrix. Overall, these findings provide preliminary chemical and biochemical evidence supporting the potential functional value of polyphenol-enriched *M. alliacea* flower extracts. Future studies employing high-resolution tandem mass spectrometry (HRMS/MS), bioactivity-guided fractionation, pharmacokinetic evaluation, and appropriate *in vivo* models are warranted to identify the constituents contributing to the observed activities and to further establish their biological and translational relevance.

## CRediT authorship contribution statement

**Quoc Trung Nguyen:** Writing – original draft, Validation, Supervision, Methodology, Formal analysis. **Tran Chi Linh:** Writing – review & editing, Writing – original draft, Validation, Supervision, Resources, Methodology, Formal analysis.

## Ethics statement

All procedures involving animals were conducted in accordance with institutional guidelines and were approved by the Institutional Ethics Committee in Biomedical Research of Nam Can Tho University under Approval No. 40YSH/PCT-HĐĐĐ.

## Institutional review board statement

Not applicable.

## Declaration of generative AI and AI-assisted technologies in the writing process

None.

## Funding

This research received no external funding.

## Declaration of competing interest

The authors declare that they have no known competing financial interests or personal relationships that could have appeared to influence the work reported in this paper.

## Data Availability

Data will be made available on request.

## Abbreviations

OMAE: Optimized *Mansoa alliacea* extract
UMAE: Unoptimized *Mansoa alliacea* extract
GAE: Gallic acid equivalents
DPPH: 2,2-Diphenyl-1-picrylhydrazyl
ABTS: 2,2′-Azino-bis(3-ethylbenzothiazoline-6-sulfonic acid)
BSA: Bovine serum albumin
RBC: Red blood cell
DMSO: Dimethyl sulfoxide
IC_50_: Half maximal inhibitory concentration
UAE: Ultrasound-assisted extraction
ANOVA: Analysis of variance
CV: Coefficient of variation
pNPG: *p*-Nitrophenyl-*α-**d**-*glucopyranoside
