# Supplementary material for: Optimization of ultrasound-assisted extraction of polyphenols from Mansoa alliacea flowers and evaluation of their antioxidant, anti-inflammatory-related, and digestive enzyme inhibitory activities
Source: Biochem Biophys Rep. 2026 Oct 31;48:102851. doi: 10.1016/j.bbrep.2026.102851 (PMC13744347; doi:10.1016/j.bbrep.2026.102851)
Supplement: Supplementary file 1 [file mmc1.docx]

**S. Figure 1.** Complete assignment (-)ESI-MS spectrum of **compound 6**

**S. Figure 2.** UV–Vis absorption spectrum of **compound 6**

**S. Figure 3.** Complete assignment (-)ESI-MS spectrum of **compound 7**

**S. Figure 4.** UV–Vis absorption spectrum of **compound 7**
